# Supplementary material for: A study of roflumilast treatment on functional and structural changes in hippocampus in depressed Adult male Wistar rats
Source: PLoS One. 2024 Feb 5;19(2):e0296187. doi: 10.1371/journal.pone.0296187 (PMC10843119; doi:10.1371/journal.pone.0296187)
Supplement: Attachment — Submitted filename: PONE-D-23-15624_reviewer-NEW 3.8.pdf [file pone.0296187.s002.pdf]

PLOS ONE

A Study of Roflumilast Treatment on Functional and Structural Changes in Hippocampus in Depressed Adult Male Wistar Rats

--Manuscript Draft--

**Manuscript Number:**	PONE-D-23-15624
**Article Type:**	Research Article
**Full Title:**	A Study of Roflumilast Treatment on Functional and Structural Changes in Hippocampus in Depressed Adult Male Wistar Rats
**Short Title:**	Effect of Roflumilast Treatment on the hippocampus in depressed male rats
**Corresponding Author:**	Ghida Mohamed Hassan<br>Ain Shams University Faculty of Medicine<br>Al-Qāhirah (incl. Ḥulwān), Al-Qahirah [Cairo] EGYPT
**Keywords:**	depression; Hippocampus; Inflammation; Oxidative stress; Roflumilast
**Abstract:**	Depression is a common stress disability disorder that affects higher mental functions including emotion, cognition, and behavior. It may be mediated by inflammatory cytokines that interfere with neuroendocrine function, and synaptic plasticity. Therefore, reductions in inflammation might contribute to treatment response. The current study aims to evaluate the role of Protein Kinase (PKA)- cAMP response element-binding protein (CREB)- brain derived neurotropic factor<br>(BDNF) signaling pathway in depression and the effects of roflumilast (PDE4 inhibitor) as potential antidepressant on the activity of the PKA-CREB-BDNF signaling pathway, histology, and pro-inflammatory cytokine production. 40 Adult male Wistar rats were divided into 4 groups:<br>Control group, Positive Control group: similar to the controls but received Roflumilast (3 mg / kg / day) by oral gavage for the last 4 weeks of the experiment, Depressed group which were exposed to chronic stress for 6 weeks, and Roflumilast-treated group which were exposed to chronic stress for 6 weeks and treated by Roflumilast (3 mg / kg / day) by oral gavage for the last 4 weeks of the experiment. The depressed group showed significant increase in immobility time with significant decrease in swimming and struggling times, significant decrease in hippocampal PKA, CERB, BDNF, Dopamine, Cortisone, and Superoxide dismutase while hippocampal Phosphodiesterase-E4, Interleukin-6, and Malondialdhyde levels were significantly elevated. These findings were significantly reversed upon Roflumilast treatment. Therefore, it could be concluded that depression is a neurodegenerative inflammatory disease and oxidative stress plays a key role in depression. Roflumilast treatment attenuated the depression behavior in rats denoting its neuroprotective, anti-apoptotic, and anti-inflammatory effects.
**Order of Authors:**	Ghida Mohamed Hassan
	Sherif A Kamar, Assistant professor
	Hagar Yousry Rady, Assistant professor
	Dina Sayed Abdelrahim, Lecturer
	Nesma Hussein Abdel Hay Ibrahim, Lecturer
	Noha N Lasheen, Assistant professor
**Additional Information:**	
**Question**	**Response**
**Financial Disclosure**<br><br>Enter a financial disclosure statement that describes the sources of funding for the work included in this submission. Review the submission guidelines for detailed	The authors received no specific funding for this work

Powered by Editorial Manager® and ProduXion Manager® from Aries Systems Corporation

requirements. View published research articles from *PLOS ONE* for specific examples.<br><br>This statement is required for submission and **will appear in the published article** if the submission is accepted. Please make sure it is accurate.<br><br>**Unfunded studies**<br>Enter: *The author(s) received no specific funding for this work.*<br><br>**Funded studies**<br>Enter a statement with the following details:<br>• Initials of the authors who received each award<br>• Grant numbers awarded to each author<br>• The full name of each funder<br>• URL of each funder website<br>• Did the sponsors or funders play any role in the study design, data collection and analysis, decision to publish, or preparation of the manuscript?<br>• **NO** - Include this sentence at the end of your statement: *The funders had no role in study design, data collection and analysis, decision to publish, or preparation of the manuscript.*<br>• **YES** - Specify the role(s) played.<br><br>* typeset	
**Competing Interests**<br><br>Use the instructions below to enter a competing interest statement for this submission. On behalf of all authors, disclose any competing interests that could be perceived to bias this work—acknowledging all financial support and any other relevant financial or non-financial competing interests.<br><br>This statement is required for submission and **will appear in the published article** if the submission is accepted. Please make sure it is accurate and that any funding sources listed in your Funding Information later in the submission form are also declared in your Financial Disclosure	The authors have declared that no competing interests exist

Powered by Editorial Manager® and ProduXion Manager® from Aries Systems Corporation

statement.<br><br>View published research articles from *PLOS ONE* for specific examples.<br><br>**NO authors have competing interests**<br><br>Enter: *The authors have declared that no competing interests exist.*<br><br>**Authors with competing interests**<br><br>Enter competing interest details beginning with this statement:<br><br>*I have read the journal's policy and the authors of this manuscript have the following competing interests: [insert competing interests here]*<br><br>* typeset	
**Ethics Statement**<br><br>Enter an ethics statement for this submission. This statement is required if the study involved:<br><br>• Human participants<br>• Human specimens or tissue<br>• Vertebrate animals or cephalopods<br>• Vertebrate embryos or tissues<br>• Field research<br><br>Write "N/A" if the submission does not require an ethics statement.<br><br>General guidance is provided below. Consult the submission guidelines for detailed instructions. **Make sure that all information entered here is included in the Methods section of the manuscript.**	The experimental protocol was approved by Physiology Department and by Ain Shams University institutional animal care unit committee with approval number FMASU R 133/2022.<br>On the day of sacrifice, overnight fasted rats were anaesthetized with intraperitoneal injection of Pentobarbitone (40 mg/kg body weight) with booster doses as needed. The dose was given according to the guideline of rodent anesthesia analgesia formulary-UBC animal care

**Format for specific study types**<br><br>**Human Subject Research (involving human participants and/or tissue)**<br>• Give the name of the institutional review board or ethics committee that approved the study<br>• Include the approval number and/or a statement indicating approval of this research<br>• Indicate the form of consent obtained (written/oral) or the reason that consent was not obtained (e.g. the data were analyzed anonymously)<br><br>**Animal Research (involving vertebrate animals, embryos or tissues)**<br>• Provide the name of the Institutional Animal Care and Use Committee (IACUC) or other relevant ethics board that reviewed the study protocol, and indicate whether they approved this research or granted a formal waiver of ethical approval<br>• Include an approval number if one was obtained<br>• If the study involved *non-human primates*, add *additional details* about animal welfare and steps taken to ameliorate suffering<br>• If anesthesia, euthanasia, or any kind of animal sacrifice is part of the study, include briefly which substances and/or methods were applied<br><br>**Field Research**<br><br>Include the following details if this study involves the collection of plant, animal, or other materials from a natural setting:<br>• Field permit number<br>• Name of the institution or relevant body that granted permission	
**Data Availability**<br><br>Authors are required to make all data underlying the findings described fully available, without restriction, and from the time of publication. PLOS allows rare exceptions to address legal and ethical concerns. See the PLOS Data Policy and FAQ for detailed information.	Yes - all data are fully available without restriction

A Data Availability Statement describing where the data can be found is required at submission. Your answers to this question constitute the Data Availability Statement and **will be published in the article**, if accepted.<br><br>**Important:** Stating 'data available on request from the author' is not sufficient. If your data are only available upon request, select 'No' for the first question and explain your exceptional situation in the text box.<br><br>Do the authors confirm that all data underlying the findings described in their manuscript are fully available without restriction?	
**Describe where the data may be found in full sentences. If you are copying our sample text, replace any instances of XXX with the appropriate details.**<br><br>• If the data are **held or will be held in a public repository**, include URLs, accession numbers or DOIs. If this information will only be available after acceptance, indicate this by ticking the box below. For example: *All XXX files are available from the XXX database (accession number(s) XXX, XXX.).*<br>• If the data are all contained **within the manuscript and/or Supporting Information files**, enter the following: *All relevant data are within the manuscript and its Supporting Information files.*<br>• If neither of these applies but you are able to provide **details of access elsewhere**, with or without limitations, please do so. For example:<br><br>*Data cannot be shared publicly because of [XXX]. Data are available from the XXX Institutional Data Access / Ethics Committee (contact via XXX) for researchers who meet the criteria for access to confidential data.*<br><br>*The data underlying the results presented in the study are available from (include the name of the third party*	All relevant data are within the manuscript and its Supporting Information files.

and contact information or URL).<br>• This text is appropriate if the data are owned by a third party and authors do not have permission to share the data.<br><br>* typeset	
Additional data availability information:	

Manuscript

Click here to access/download;Manuscript;Effect of Roflumilast on the hippocampus in rats.docx

A Study of Roflumilast Treatment on Functional and Structural Changes in Hippocampus in Depressed Adult Male Wistar Rats

***Authors list and affliations**

Ghida Hassan[1], Sherif A. Kamar[2], Hagar Yousry Rady[2], Dina Sayed Abdelrahim[3], Nesma Hussein Abdel Hay Ibrahim[4], Noha N Lasheen[5]

1: Lecturer, Medical Physiology department, Faculty of Medicine, Ain Shams University, Cairo, Egypt.

2: Assistant Professor, Anatomy department, Faculty of Medicine, Ain Shams University, Cairo, Egypt.

3: Lecturer, Clinical Pharmacology department, Faculty of Medicine, Ain Shams University, Cairo, Egypt.

4: Lecturer of medical biochemistry and molecular biology, Faculty of Medicine, Ain Shams University, Cairo, Egypt.

5: Assistant Professor of Medical Physiology, Faculty of Medicine, Ain Shams University and Galala University, Egypt.

***Corresponding author:**

Ghida Hassan

email: ghidamh.55@med.asu.edu.eg

Telephone number:01001654547

Orcid ID: 0000-0001-5659-1610

Abstract:

Depression is a common stress disability disorder that affects higher mental functions including emotion, cognition, and behavior. It may be mediated by inflammatory cytokines that interfere with neuroendocrine function, and synaptic plasticity. Therefore, reductions in inflammation might contribute to treatment response. The current study aims to evaluate the role of Protein Kinase (PKA)- cAMP response element-binding protein (CREB)- brain derived neurotropic factor (BDNF) signaling pathway in depression and the effects of roflumilast (PDE4 inhibitor) as potential antidepressant on the activity of the PKA-CREB-BDNF signaling pathway, histology, and pro-inflammatory cytokine production. Forty Adult male Wistar rats were divided into 4 groups:

Control group, Positive Control group: similar to the controls but received Roflumilast (3 mg / kg / day) by oral gavage for the last 4 weeks of the experiment, Depressed group which were exposed to chronic stress for 6 weeks, and Roflumilast-treated group which were exposed to chronic stress for 6 weeks and treated by Roflumilast (3 mg / kg / day) by oral gavage for the last 4 weeks of the experiment. The depressed group showed significant increase in immobility time with significant decrease in swimming and struggling times, significant decrease in hippocampal PKA, CERB, BDNF, Dopamine, Cortisone, and Superoxide dismutase while hippocampal Phosphodiesterase-E4, Interleukin-6, and Malondialdhyde levels were significantly elevated. These findings were significantly reversed upon Roflumilast treatment. Therefore, it could be concluded that depression is a neurodegenerative inflammatory disease and oxidative stress plays a key role in depression. Roflumilast treatment attenuated the depression behavior in rats denoting its neuroprotective, anti-apoptotic, and anti-inflammatory effects.

Keywords:

Depression, Hippocampus, Inflammation, Oxidative stress, Roflumilast

Introduction:

Depression is a widespread condition that affects people of all sexes, ages, socioeconomic classes, and species. According to the World Health Organization, depression is considered the main factor contributing to disability. In addition to feelings of guilt, poor focus, low self-esteem, sleep difficulties, and disturbed appetite, it also manifests as a lack of interest, desire, and pleasure. **(1).**

Stress hormones are consistently over-secreted in depression, which leads to severe clinical manifestations and inhibits a sufficient response to stress. These hormones influence higher mental functions including emotion, cognition, and behaviour in the brain by acting as neuromodulators. An important risk factor for making someone susceptible to stress-related diseases is chronic stress, which increases the production of corticosteroids and affects corticosteroid-receptor signalling (**2**). According to a subsequent research, depression is a complex condition with many underlying causes and has been linked to an increased chance of developing serious medical conditions, including diabetes, cancer, Alzheimer's disease, epilepsy, cardiovascular disease, and stroke (**1**).

It was hypothesised that chronic stress might suppress neurogenesis, retract dendritic processes, and cause neuronal death in the hippocampus since research on people subjected to chronic stress revealed decreased hippocampal sizes (**3**).

Additionally, depression may cause disturbances in the way the brain regulates glucose and may be linked to cognitive decline. Leptin levels, negative mood, and sleep disruptions were revealed to be strongly positively correlated with one another (**4**). Rats' ventral striatum's basal and feeding-stimulated dopamine (DA) release can both be reduced by leptin (**5**). Furthermore, long-

term inhibition of leptin signalling in the ventral tegmental area promotes locomotor activity whereas activation of leptin receptors suppresses firing of ventral tegmental area DA neurons (**6**). Major depression may also result from an imbalance of ghrelin feedback in brain areas responsible for mood regulation. It has been discovered that ghrelin controls mood, dopaminergic characteristics, and the growth of the central nervous system. In both mice and people, it has antidepressant effects that have an impact on rewarding behaviour (**7**).

Acute phase proteins, such as C-reactive protein and haptoglobin, as well as inflammatory cytokines like interleukin-1 (IL-1), interleukin-6 (IL-6), and TNF-alpha were shown to be typically related with illness behaviour and depression, respectively. Such inflammatory cytokines can interact with any mediator associated with depression, including synaptic plasticity, neurotransmitter metabolism, and neuroendocrine function. Accordingly, inflammatory alterations may play a role in the development of depressive disorders, and as a result, decreasing inflammation may improve treatment outcomes (**8**).

It has been established that brain monoamines including norepinephrine (NE), serotonin (5-HT), and dopamine (DA), as well as their metabolites, play a role in the etiology of depression. Therefore, NE, 5-HT, and DA neurotransmitter deficits aggravate depressive diseases (**9**).

Numerous tools were examined, such as the Protein Kinase CAMP-Initiated Reactant Subunit Alpha (PRKACA) overflow, a significant cell signalling pathway that may be linked to the onset of depression by regulating synaptic pliancy, cytokinesis, transcriptional guidance, and the hypothalamic-pituitary-adrenal (HPA) axis (**10**). Additionally, CREB is a transcriptional regulator that controls the expression of brain derived neurotropic factor (BDNF), a significant biomarker of depression (**11**), which is crucial for neuronal survival and function (**12**). Recently, it was discovered that CREB/BDNF expression is essential for the pathophysiology of depression (**13**).

The chronic restraint stress (CRS) model is a well-established model for producing depressive-like behavior in animals (**14**). It's interesting to note that CRS results in long-lasting neuroinflammation, which is a key pathophysiologic cause of severe depressive disorders (**15**). Furthermore, it was discovered that CRS reduced BDNF levels (**16**). However, in the hippocampus and cortex of mammals, phosphodiesterase 4 (PDE4) is produced and selectively hydrolyzes cAMP to 5′-AMP. In recent years, it has been speculated as a possible depression drug target (**17**). PDE4 inhibitors have been reported to reduce symptoms similar to depression. The PDE4 inhibitors that have currently been discovered include rolipram, roflumilast, ibudilast, MK-0952, HT-0712, GEBR-32a, and FFPM. It has been established that roflumilast plays a part in both synaptic plasticity and learning memory (**18**). Roflumilast was discovered to enhance synaptic plasticity and memory formation in rats (**19**) and to lessen depression-like behaviour in mice with Alzheimer disease (**20**). According to these studies, roflumilast may one day be used as an antidepressant.

Aim of the work:

This study investigated the effects of roflumilast (PDE4 inhibitor) on the activity of the PRKACA-CREB-BDNF signaling pathway, pro-inflammatory cytokine production and change in hippocampal histology in male Wistar rats exposed to CRS model of depression. This study demonstrated the PKA-CREB-BDNF signaling pathway role in controlling stress-induced microenvironmental changes connected to a variety of neurological disorders, including depression.

Materials and Methods:

Ethics approval

All animal protocols complied with the Guide for the Care and Use of Laboratory Animals published by the US National Institutes of Health (NIH Publication No. 85–23, revised 2011) and were conducted with the approval of Physiology Department and by Faculty of Medicine-Ain Shams University research ethics committee, the approval number is **FMASU R 133/2022.**

Animals

This study was performed on Forty adult Wistar male rats weighing between 150 and 200 g, purchased from the Holding Company for Biological Products and Vaccines (VACCERA), Helwan, Egypt. In animal house of Department of Pharmacology, Faculty of Medicine, Ain Shams University, animals were kept in plastic cages with enough ventilation, a temperature range of 22–25 °C, a 12-hour light/dark cycle, and free access to food and drink. To lessen the possibility of animal distress, the animals were allowed to adapt to the new surroundings for 7 days prior to experimental treatments. Animals were not subjected to unneeded suffering or stress, and all handling were done with the utmost care and cleanliness.

Rats were randomly divided into four groups:

- Control group (n=10); which were fed balanced chow diet-ad libitum-for 6 weeks.

- Positive control group (n=10); which were similar to the controls but received Roflumilast (WESTABREATH 500 ®) 3 mg / kg / day, by oral gavage, dissolved in distilled water for the last 4 weeks of the experiment ***(*21).**

- **Depressed group (n=10)**, which were exposed to chronic restraint stress for 6 weeks. The stress procedure was carried out in a different room by using a mild stress, restraint, which consisted of placing rats in Plexiglas tubes (25 x 8 cm) wide enough to allow comfortable breathing but restricting their movement, for 4 hours per day for 6 weeks performed at fixed time. Every stress session was carried out between 9:00 AM and 1:00 PM **(22)**.

- **Roflumilast-treated group (n=10):** rats were exposed to chronic restraint stress 4 hours per day for 6 weeks and were treated by Roflumilast (WESTABREATH 500 ®) 3 mg / kg / day, by oral gavage, dissolved in distilled water for the last 4 weeks of the experiment **(21)**.

Behavioral tests:

At the end of 6 weeks, the experimental period, all animals were subjected to behavioral tests including Open Field Test (OFT) and Forced Swimming Test (FST) **(23).**

Open Field Test (OFT):

Rats were given one hour to get used to the test room before the test began. Each rat was left alone for five minutes in the middle of a quadrangular arena (60x60 cm, divided into 16 identical squares) that was well-lit. The following characteristics were determined: the number of squares traversed (with all four feet crossed), the amount of time spent in the central zone (central four squares), the frequency of entry, and the latency to leave the central zone. After each rat, the OFT arena was cleaned with 10% alcohol **(23)**.

Forced Swimming Test (FST):

Rats swam in water that was 35 cm deep during FST in a vertical Plexiglass cylinder that was 22 cm in diameter and 50 cm in height. The water was kept at a constant 25 °C temperature. The rats swam for 15 minutes on the first day of training. On the next day (test day), rats swam for five minutes. Calculations of the immobility, swimming, and struggling times were made. Each rat underwent the test just once. Following each animal's test, the water was replaced **(23)**.

Sacrifice and sample collection:

On the day of sacrifice, overnight fasted rats were anaesthetized with intraperitoneal injection of Pentobarbitone (40 mg/kg body weight) with booster doses as needed. The dose was given according to the guideline of rodent anesthesia analgesia formulary-UBC animal care **(24)**.

When the stage of surgical anesthesia (judged by loss of withdrawal reflexes) had been reached, the animal was placed on its back and fixed on the operating table. The rat skull was opened, and the brain was dissected and washed by saline. The hippocampus was reached from the medial side after division of the brain at the mid sagittal plane into right and left hemispheres, followed by removal of the whole brain stem and cerebellum **(25)**.

The hippocampus from all studied groups were stored at -80°C for subsequent biochemical studies:

1-Reverse Transcription Polymerase Chain Reaction (RT-PCR) method for PDE-4 and phosphorylated CERB determination

RNA extraction:

Total RNA was extracted from homogenized tissues of all different groups using Direct-zol RNA Miniprep Plus (Cat# R2072, ZYMO RESEARCH CORP. USA) and then quantity and quality were assessed by Beckman dual spectrophotometer (USA).

Real time PCR:

SuperScript IV One-Step RT-PCR kit (Cat# 12594100, Thermo Fisher Scientific, Waltham, MA USA) was utilized for reverse transcription of extracted RNA followed by PCR. 96-well plate StepOne instrument (Applied Biosystem, USA) was used in a thermal profile as follows: 10 minutes at 45 ºC for reverse transcription, 2 minutes at 98 ºC for RT inactivation and initial denaturation by 40 cycles of 10 seconds at 98ºC, 10 seconds at 55 ºC and 30 second at 72 ºC for the amplification step. After the RT-PCR run the data were expressed in Cycle threshold (Ct) for the target genes and housekeeping gene. Normalization for variation in the expression of target genes; *PDE-4, and CREB* were performed referring to the mean critical threshold (CT) expression values of *GAPDH* housekeeping gene by the ΔΔCt method. The relative quantitation (RQ) of each

target gene is quantified according to the calculation of $2^{-\Delta\Delta Ct}$ method. Primers sequence for *PDE-4* gene was; forward 5′- GCTTGAACACCAACGTCCCACGGT -3′, and reverse 5`- GCTGAGGTTCTGGAAGATGTCGCAG -3′ (gene bank accession number NM_013101.4), *CREB* gene was; forward 5′- GAGACCTGGCCAGAGGATAC -3′, and reverse 5`- GTCAGTGAGCAAGAGAACGC -3′ (gene bank accession number NM_001005562.2), and *GAPDH* housekeeping gene was; forward 5'- CCTCGTCTCATAGACAAGATGGT -3' and reverse 5'- GGGTAGAGTCATACTGGAACATG -3' (gene bank accession number NM_001394060.2).

2-Measurements of proteins by enzyme-linked immunosorbent assay (ELISA) in hippocampus tissues:

Preparation of tissue homogenate

Animals after they were sacrificed, tissues were washed thoroughly and rinsed with ice. They were gently blotted between the folds of a filter paper and weighed in an analytical balance. 10% of homogenate was prepared in 0.05 M phosphate buffer (pH 7) using a polytron homogenizer at 4^0C. The homogenate was centrifuged at 10,000 rpm for 20 min for removing the cell debris, unbroken cells, nuclei, erythrocytes and mitochondria. The supernatant (cytoplasmic extract) was used according to manual instructions.

Determination of tissue protein

Protein content in the tissue was determined using Genei, Bangalore, protein estimation kit. **(26)**

All ELISA kits were measured by ELISA reader. Color absorbance was read at OD range 490 to 630 nm using an Enzyme-Linked Immuno-Sorbent Assay (ELISA) plate reader (Stat Fax 2200, Awareness Technologies, Florida, USA).

Protein Kinase CAMP-Activated Catalytic Subunit Alpha (PRKACA) was measured using Rat Protein Kinase A ELISA Kit (Bio-assay technology laboratory), the concentration of BDNF, IL-6 in tissues (pg/mg) were measured using Enzyme-linked Immunosorbent Assay Kit for BDNF and IL- 6 (Cloud Cone Corporation). DA and Corticosterone (CORT) were measured using Mouse / Rat Dopamine ELISA Assay Kit (Eagle Bioscience, Catalog No, DOU39-K01) and Rat cortisol ELISA Kit (CUSABIO, Catalog No, CSB-E05112r) respectively.

Measurement of oxidative stress markers:

The activities of SOD (U/mg) & MDA (nmol/mg) were determined by using Superoxide Dismutase (SOD) Activity Assay Kit (BioVision, USA, Catalog #K335), and Lipid Peroxidation (MDA) Colorimetric/Fluorometric (BioVision, USA, Catalog # K739) respectively.

(II) Histological studies:

Histological studies:

The brain hemispheres were processed for paraffin blocks. Serial parasagittal sections were done. The sections were stained with hematoxylin and eosin **(27)**. Other specimens 1mm^3 were immediately fixed in 4% glutaraldehyde and processed for semithin sections. Sections were stained with Toluidine blue **(28).** The sections were examined with an Olympus light microscope (CX31) equipped with digital camera in Anatomy department and were photographed.

GFAP immunohistochemistry was done to demonstrate the astrocytes. Sections were Deparaffinated, hydrated and incubated in blocking solution TBT [Tris Base Saline (TBS), 0.5 M pH 7.4 containing 3% (w/v) bovine serum albumin and 0.05% (v/v) Triton X-100]. Sections were incubated overnight at 4 °C in a humidified chamber with the anti- glial fibrillary acidic protein (GFAP) mouse monoclonal antibody at a 1:50 dilution. Slides were washed for 5 min in TBS. Immunodetection was performed using biotinylated anti-mouse immunoglobulins then incubation

with 3,3 diaminobenzidine (DAB) chromogen (DAKO) in hydrogen peroxide for 5-10 minutes (brown staining). The sections were lightly counterstained with Mayer's hematoxylin, dehydrated and mounted **(29).**

Statistical analysis

All results in this study were expressed as mean ± SEM. Statistical Package for the Social Sciences (SPSS, Inc., Chicago, IL, USA) program, version 20.0 was used to compare significance between groups. One Way ANOVA (Analysis Of Variance) for difference between means of different groups was performed on the results obtained in the present study. Differences were considered significant when P is ≤ 0.05.

Results:

Behavioral tests, FST:

As regards FST, immobility time was significantly elevated, meanwhile, swimming and struggle times were significantly lowered in untreated depressed groups compared to the controls ($P\leq0.001$, $P\leq0.001$, $P\leq0.001$ respectively). However, immobility time was significantly lowered, swimming time was significantly elevated in treated depressed group compared to the untreated depressed rats ($P\leq0.001$, $P\leq0.001$ respectively). Immobility and struggle times were significantly elevated in positive control rats, while swimming time was significantly lowered when compared to the control group ($P\leq0.001$, $P\leq0.001$, $P\leq0.001$ respectively), as shown in figure 1.

Behavioral tests, OFT

As regards OFT, total number of crossed squares was significantly decreased, meanwhile, time spend in the central zone and latency to leave the central zone were significantly increased in untreated depressed groups compared to the controls ($P\leq0.001$, $P\leq0.05$, $P\leq0.001$ respectively). However, total number of crossed squares was significantly elevated, latency to leave the central

zone was significantly lowered in treated depressed group compared to the untreated depressed rats (P≤0.001, P≤0.01 respectively). The total number of crossed squares and the latency to leave the central zone were significantly lowered in positive control rats, when compared to the control group (P≤0.001, P≤0.05 respectively), as shown in figure 1.

Fig (1): Behavioral tests changes in in the different studied

Changes in PRKACA -CREB-BDNF signaling pathway:

Regarding hippocampal PKA, CERB and BDNF levels were significantly lowered in untreated depressed group compared to the control group (P≤0.001 for each). they were significantly elevated in Roflumilast-treated depressed group compared to the untreated depressed rats (P≤0.001 for each). Non-significant changes were present in positive controls when compared to negative control ones, as shown in figure 2.

Fig (2): Changes in Hippocampal levels of PRKACA, CERB, and BDNF in the different studied groups.

Changes in PDE4 and IL-6:

Meanwhile, hippocampal PDE4 and IL-6 levels, they were significantly elevated in untreated and Roflumilast-treated depressed groups compared to the controls (P≤0.001, P≤0.05, P≤0.001, P≤0.05 respectively). However, they were significantly lowered in Roflumilast-treated depressed group compared to the untreated depressed ones (P≤0.001 for both). No significant differences were observed in positive controls when compared to negative control rats, as shown in figure 2 and 3.

Fig (2) & Fig (3): Changes in Hippocampal levels of phosphodiesterase 4 and IL6 in the different studied groups.

Changes in DA and CORT:

Both hippocampal DA and CORT were significantly decreased in untreated depressed group compared to the controls ($P \leq 0.001$ for each), meanwhile, both were significantly increased in Roflumilast-treated depressed group compared to the untreated depressed rats ($P \leq 0.001$ for each), as shown in figure 3.

Fig (3): Changes in Hippocampal levels of dopamine and cortisone in the different studied groups.

Oxidative Stress Markers Changes:

As shown in figure 4, the untreated depressed group had significant higher hippocampal MDA level and significant lower hippocampal SOD level when compared to control ones ($P \leq 0.001$ for each), while Roflumilast-treated depressed rats had significantly lowered hippocampal MDA and significantly elevated hippocampal SOD compared to the untreated depressed rats ($P \leq 0.001$, for each). Non-significant differences were observed when comparing positive controls and negative control rats.

Fig (4): Oxidative stress markers changes in in the different studied groups.

Histological Results:

Histological examination of both the control and positive control groups showed almost the same results in all the examined stained sections, so the selected photomicrographs of the control group will be also representing for the positive control group.

- **<u>H&E-stained sections:</u>**

Examination of the H&E-stained sections of CA1 region of the group I (Control group) showed that the hippocampal formation was formed of two regions; the hippocampus proper (Cornu Ammonis, with its four regions; CA1, CA2, CA3 and CA4) and the dentate gyrus (DG) (Fig. 5).

The cells forming the CA1 region were arranged in three layers: the polymorphic layer, the pyramidal cell layer and the molecular layer. The pyramidal cell layer appeared to be the principal layer of the hippocampus proper and was formed of pyramidal cells with large rounded vesicular nuclei and prominent nucleoli. Both the polymorphic and the molecular layers showed scanty scattered glial cells (Fig. 6).

Fig (5): A photomicrograph of a section of the hippocampus of an adult male rat (control group) showing the two regions of the hippocampal formation; the hippocampus proper (Cornu ammonis with its four regions; CA1, CA2, CA3 and CA4) and the dentate gyrus (DG). **(H&E x 100)**

Fig (6): A photomicrograph of a section of the CA1 region of the hippocampus of a rat of group I (control group) showing the pyramidal layer (Pr) formed of pyramidal cells with large rounded vesicular nuclei **(black arrow**) and prominent nucleoli **(red arrow).** Notice the apical and basal processes of the pyramidal cells **(yellow arrow).** Note that both the molecular layer (Mo) and the polymorphic layer (Po) show scanty scattered glial cells **(Circle). (H&E × 400)**

Examination of the H&E-stained sections of CA1 region of group III (Depressed group) revealed that most of the pyramidal cells had irregular outlines with dark deeply stained nuclei with pericellular spaces (vacuolations) around some of the pyramidal cells. Also, pericellular vacuolation around the glial cells and spacing within the molecular layer and the polymorphic layers were detected (Fig. 7).

Fig (7): A photomicrograph of a section of the CA1 region of the hippocampus of a rat of group III (Depressed group) showing most of the pyramidal cells have irregular outlines with dark deeply stained nuclei (**yellow arrow**). Notice the pericellular spaces (vacuolations) **(V)** around some of the pyramidal cells. Also notice the pericellular vacuolations around the glial cells and spacing **(V)** within the molecular layer and the polymorphic layers. Mo=molecular layer, Pr= pyramidal layer, Po= polymorphic layer. **(H&E × 400)**

Examination of the H&E-stained sections of CA1 region of group IV (Roflumilast-treated group) showed that most of the pyramidal cells had large rounded vesicular nuclei with prominent nucleoli. Few pyramidal cells showed irregular outlines and variable sizes with darkly stained nuclei and pericellular spaces. Pericellular vacuolation around the glial cells and spacing within the molecular layer and the polymorphic layers were detected (Fig. 8).

Fig (8): A photomicrograph of a section of the CA1 region of the hippocampus of a rat of group IV (Roflumilast-treated group) showing that most of the pyramidal cells within the pyramidal layer (Pr) have large rounded vesicular nuclei (**black arrow**) with prominent nucleoli (**red arrow**). Few darkly stained pyramidal cells with irregular outlines and variable sizes (**yellow arrow**) can be seen. Notice the presence of pericellular spaces around few of the pyramidal cells (**V**). Also, notice the pericellular vacuolations around the glial cells and spacing **(V)** within the molecular layer (Mo) and the polymorphic layers (Po). **(H&E × 400)**

- **<u>Toluidine blue stained semithin sections:</u>**

Examination of the toluidine blue stained semithin sections of CA1 region of group I (Control group) showed that the pyramidal layer was formed of densely packed pyramidal cells with large rounded vesicular nuclei and prominent nucleoli with projecting processes. The presence of glial cells with pale ovoid nuclei and lightly stained cytoplasm was detected within the molecular and polymorphic layers (Fig. 9).

Fig (9): A photomicrograph of a semithin section of the CA1 region of the hippocampus of a rat of group I (control group) showing the pyramidal layer (Pr) formed of densely packed pyramidal cells with large rounded vesicular nuclei **(black arrow**), prominent nucleoli **(red arrow)** and projecting processes **(yellow arrow).** Notice the presence of glial cells **(g)** with pale ovoid nuclei and lightly stained cytoplasm**. (Toluidine blue x 1000)**

Examination of the toluidine blue stained semithin sections of CA1 region of group III (Depressed group) showed the pyramidal cells with irregular outlines, variable sizes and with dark deeply stained nuclei. Many glial cells with pale ovoid nuclei and lightly stained cytoplasm throughout the layers were also detected (Fig. 10).

Fig (10): A photomicrograph of a semithin section of the CA1 region of the hippocampus of a rat of group III (Depressed group) showing the pyramidal cells having irregular outlines and variable sizes with dark deeply stained nuclei (arrow). Notice the presence of many glial cells (g) with pale ovoid nuclei and lightly stained cytoplasm throughout the layers. Mo= molecular layer, Pr= pyramidal layer and Po= polymorphic layer. **(Toluidine blue x 1000)**

Examination of the toluidine blue stained semithin sections of CA1 region of group IV (Roflumilast-treated group) revealed that most of the pyramidal cells within the pyramidal layer appeared densely packed with large rounded vesicular nuclei, prominent nucleoli and with projecting processes. Very few pyramidal cells had dark deeply stained nuclei with irregular outline. Few glial cells with pale ovoid nuclei and lightly stained cytoplasm could be seen within the within the molecular and polymorphic layers (Fig. 11).

Fig (11): A photomicrograph of a semithin section of the CA1 region of the hippocampus of a rat of group IV (Roflumilast-treated group) showing that most of the pyramidal cells within the pyramidal layer (Pr) appear densely packed with large rounded vesicular nuclei **(black arrow**), prominent nucleoli **(red arrow)** and projecting processes **(yellow arrow).** Very few pyramidal cells have dark deeply stained nuclei with irregular outline **(dotted arrow**). Notice the presence of a few glial cells **(g)** with pale ovoid nuclei and lightly stained cytoplasm. Mo=molecular layer and Po= polymorphic layer. **(Toluidine blue x 1000)**

➢ **Immunohistochemical (GFAP) stained sections:**

Examination of the GFAP stained sections of CA1 region of group I (Control group) showed that apparent minimal GFAP immunostaining of the astrocytes represented by minimal brownish color (Fig. 12).

Fig (12): A photomicrograph of a section of CA1 region of the hippocampus of a rat of group I (Control group) showing apparent minimal GFAP immunostaining of the astrocytes represented by minimal brownish color. MO = molecular layer, Pr = pyramidal layer and Po= polymorphic layer. **(GFAP x 400)**

Examination of the GFAP stained sections of CA1 region of group III (Depressed group) showed apparent extensive GFAP immunostaining of the astrocytes with ramifying processes along the layers (Fig. 13).

Fig (13): A photomicrograph of a section of CA1 region of the hippocampus of a rat of group III (Depressed group) showing apparent extensive GFAP immunostaining of the astrocytes with ramifying processes along the layers. MO = molecular layer, Pr = pyramidal layer and Po= polymorphic layer. **(GFAP x 400)**

Examination of the GFAP stained sections of CA1 region of group IV (Roflumilast-treated group) revealed apparent minimal GFAP immunostaining of the astrocytes (Fig. 14).

Fig (14): A photomicrograph of a section of CA1 region of the hippocampus of a rat of group IV (Roflumilast-treated group) showing apparent minimal GFAP immunostaining of the astrocytes. MO = molecular layer, Pr = pyramidal layer and Po= polymorphic layer. **(GFAP x 400)**

Correlation studies:

In the current study, significant negative correlations exist in all studied groups between MDA and CERB, PRKACA, BDNF, DA and SOD while a significant positive correlation exists between MDA and PDE4, IL-6 and CORT (table 1). Also, significant negative correlations were observed between PDE4 and CERB, PRKACA, BDNF while there was a significant positive correlation exists between PDE4 and IL-6 (table 2). In addition, there were significant negative correlations between DA and the immobility time, time Spent in Central Zone, Latency to Leave Central Zone, while significant positive correlations were found between DA and swimming time, struggle time and total no. of crossed squares, as shown in table (3).

Table (1): correlations of MDA and other parameters in all studied groups.

	**CERB**	**PRKACA**	**BDNF**	**DA**	**SOD**	**PDE4**	**IL-6**	**CORT**
**R**	-0.65	-0.72	-0.69	-0.67	-0.72	0.66	0.78	0.64
**P**	≤0.01	≤0.001	≤0.001	≤0.001	≤0.001	≤0.001	≤0.001	≤0.01

Table (2): Correlation of PDE4 and CERB, PRKACA, BDNF and IL-6.

	**CERB**	**PRKACA**	**BDNF**	**IL-6**
**R**	-.0871	-0.894	-0.761	0.809
**P**	≤0.001	≤0.001	≤0.001	≤0.001

Table (3): Correlation of DA and findings of behavioral tests in all studied parameters

	Immobility Time	Time Spent in Central Zone	Latency To Leave Central Zone	Swimming Time	Struggle Time	Total No. Of Crossed Squares
**R**	-0.885	-0.485	-0.664	0.844	0.456	0.741
**P**	≤0.001	≤0.01	≤0.001	≤0.001	≤0.05	≤0.001

Discussion:

Significant efforts were directed toward identifying novel therapeutic targets for depression. This study assessed the effects of treatment with roflumilast on many studied neurotransmitters and mediators in hippocampi and its role in alleviation of depressive-like behavior elicited by chronic restrain stress (CRS). The present study reports CRS-induced depressive-like behavior manifested by an increase in FST immobility time with decreased times of swimming and struggling. These behavioral changes reflect the development of anhedonia and behavioral despair which are key features of human depressive symptomatology **(30).** The decreased number of crossed squares and the increased latency to leave central zone in open field test (OFT) reflect a freezing behavior which is an index of the depression-associated anxiety **(31)**. The depressive-like behavior was associated with alteration in some biochemical parameters such Protein Kinase cAMP-Activated Catalytic Subunit Alpha (PRKACA), Phosphorylated cAMP

response element-binding protein (CREB) and Brain derived neurotrophic factor (BDNF), Phosphodiesterase E4 (PDE4).

The results of this study demonstrated that roflumilast mediated reversal of depressive-like behavior induced by CRS exposure. The antidepressant action of roflumilast was demonstrated in several experimental studies. Similarly, **Wang et al. (2020)** found decreased immobility time and proved that roflumilast reversed behavioral dysfunction in APP/PS1 transgenic mice, a widely used model of Alzheimer's disease (AD). **Feng et al. (2019) (32)**, also, proved that roflumilast reversed cognition deficits and depression-like effects in APP/PS1 transgenic mice. A recent study **(33)** demonstrated that roflumilast enhanced imipramine's effect, suggesting its superior efficacy as an adjuvant to standard antidepressants. Due to its significant antidepressant effect, roflumilast was able to reduce immobility time in the previous studies. Our finding is aligned with the study of **Hasan et al. (2022) (34)**, which demonstrated that roflumilast increased rats 'exploratory potential as it increases number of crossed squares in OFT. In line, **Waltrick et al. (2023) (35)** mentioned that roflumilast was able to decrease depression-associated anxiety demonstrated by increased latency to leave central zone. Chronic hypercortisolemia results in the release of reactive oxygen species (ROS), increased NF-B transcriptional activity, and decreased synthesis of neurotropshic factors in many glucocorticoid receptor rich brain areas, such as the prefrontal cortex, amygdala, and hippocampus, with subsequent induction of structural and functional deficits. This sustained "stress responsive" HPA axis hyperactivity has been linked to the pathophysiology of depression and its associated cognitive dysfunction **(36-37)**. PDE inhibitors were also reported to modulate brain functions by altering the cAMP/cGMP/BDNF signaling **(38)**.

In the current study, Hippocampal Protein Kinase cAMP-Activated Catalytic Subunit Alpha (PRKACA), Phosphorylated cAMP response element-binding protein (CERB) and Brain derived

neurotrophic factor (BDNF) levels were significantly lowered in the untreated depressed group. However, hippocampal Phosphodiesterase E4 (PDE4) level was significantly elevated in the untreated depressed rats. These changes were found to be significantly reversed by treatment with roflumilast.

BDNF plays a crucial role in sustaining synaptic plasticity, synapse development, and neuronal proliferation. The phosphorylation of cAMP-response element-binding protein (CREB) regulates BDNF expression, which could be regarded a critical target for alleviating neuron injury induced by BDNF depletion. CREB phosphorylation is controlled by cAMP PKA. **(39*)***. PDE4 is a cyclic adenosine monophosphate (cAMP) hydrolase that uses the cAMP/PKA signaling pathway to control CREB phosphorylation. **(40).**

In the current study, stress exposure in the depressed rats resulted in decreased expression of BDNF in the hippocampus and a decrease in intracellular signaling pathways that influence BDNF expression confirmed by the significant decrease in CERB & PKA. Moreover, PDE4 was significantly increased in depressed rats reflecting its role in inducing depression.

These findings go with ***Zhong et al. (2019)*** **(41),** who mentioned that brain-derived neurotrophic factor was significantly downregulated in the hippocampal tissues of depressed rats leads to the apoptosis of neurons, in addition, low levels of BDNF were also suggested to be lower in the cerebral cortex of depressed and suicide subjects. **(42).** In support, there is a significant inverse relationship between BDNF blood levels and depression scores in depressed patients **(43)**. That is, the decrease of BDNF level in the brain is an important cause of depression.

As a result, Roflumilast, a selective inhibitor of phosphodiesterase-4 (PDE-4) that promotes BDNF expression and alleviates depression-induced neuronal injury and apoptosis, induces CREB phosphorylation. These results are in line with those of **Zhang et al. (44)**, who stated that

roflumilast affected BDNF through acting on the cAMP/CREB signaling pathway, by inhibiting PDE-4 actions. The elevated IL-6 levels in untreated and Roflumilast-treated depressed groups, despite being lowered in Roflumilast-treated depressed group compared to the untreated depressed ones could be explained by chronic stress-induced activation of inflammatory pathways triggering the development of depression, thus depressive disorders could partly be explained by some inflammatory mediators, namely increased IL-6.

Also, the higher IL-6 levels in depressed rats, herein, could be attributed to depression-induced alteration of local brain activity **(45).** Also, it was suggested that cytokines could affect brain activity through neurotransmitter-depletion, neuroendocrine changes, and neural plasticity pathways **(46).** Therefore, inflammatory cytokines were found to inhibit neurogenesis. In support, anti-inflammatory administration caused recovery or enhancement of neurogenesis **(46)**. Thus, chronic stress was found to stimulate cytokine release to blood and microglia hindering neurogenesis. In addition, depression was explained by the neurogenic theory which suggested that impaired neurogenesis could result to depression and antidepressive drugs could improve neurogenesis **(47).**

The IL-6-lowering effect of roflumilast, in the present study, could be due to its targeting effects in the neurotrophic pathway in ameliorating the depressive manifestations by lowering the neurotrophic factors, altering the plasticity and neurogenesis in the brain, thereby having anti-depressant potentials. Therefore, the reduced immobility time and the anti-depressant activity, observed in the present study, could be explained by adjusting neurotrophic pathways.

The lowered hippocampal DA, herein, in untreated depressed group despite being significantly elevated in Roflumilast-treated depressed group compared to the untreated depressed rats. This may be attributed to stress-induced downregulation of dopaminergic pathways causing depression.

In accordance, many studies **(48-49)** demonstrated that the altered function of dopaminergic system in depression in the form of lowered synaptic DA release and reduced striatal dopaminergic activity in depressed subjects.

The higher DA levels caused by Roflumilast administration, herein, may be attributed to increased DA synthesis and turnover in striatum, thereby the dopamine D1 receptor signaling is enhanced in frontal cortical pyramidal neurons **(50).**

Interestingly, hippocampal corticosteroid was significantly decreased in untreated depressed group compared to the controls, despite being significantly elevated in Roflumilast-treated depressed group compared to the untreated depressed rats. This may be attributed to altered corticosteroid level caused by stress exposure in the current study. ***Dremencov et al.*** **(51)** suggested that lowered blood corticosteroid concentrations may be due to increased metabolism and/or tissue uptake and being independent on steroidogenesis. They suggested that the decline in circulating corticosterone in rats exposed to repeated chronic stressor may be a compensatory mechanism for the higher excitability of serotonergic neurons to limit the deleterious effects of exposure to the repeated chronic stressor on the brain.

Moreover, stress-induced depressive-like behavior, herein, was associated by prominent oxidative stress denoted by higher hippocampal MDA level and lower hippocampal SOD, while Roflumilast treatment ameliorated these oxidant state changes. As previously suggested, the imbalance between reactive oxygen species (ROS) and antioxidants could damage structures and functions of lipids, proteins, and DNA. However, such changes can be ameliorated by antioxidant defense systems, such as the enzymes superoxide dismutase and catalase ***(52).***

The altered hippocampal mediators could be attributed to the evident oxidative stress which may increase the neuronal inflammatory agents and apoptotic mediators, leading to neuronal death.

In addition, the inflammatory mediators may impair the glial cells, namely astrocytes and microglia, producing neurodegenerative changes **(53),** which may explain the higher hippocampal IL-6. Moreover, oxidative stress may be the causative agent of dopaminergic neurons damage **(54).** This may explain the reduction in DA level in the current study.

These findings are in line with **Tuon et al. (55)**, who studied depressive-like behavior and oxidative stress parameters. They suggested that neurodegenerative changes, elevated lipid peroxidation, and lowered superoxide dismutase activity may produce major depressive disorders. On the other hand, Roflumilast treatment ameliorated the oxidative stress in the treated group evidenced by significant decrease in MDA and significant increase in the SOD, which are in agreement with ***Bhatt et al. (2020)*** who demonstrated that the neuroprotective effects of Roflumilast on the rat hippocampal neurons against the oxidative stress and improved the synaptic functions.

The PV1 interneuron numbers in hippocampal subdivision were proved to be reduced under the effect of long-term stress using light microscopic studies **(56-58)**. The Hilar region including PV1 neuronal degenerative changes or even necrosis were also reported in response to chronic stress **(59)**. In agreement with the current study, examination of the H&E-stained sections and toluidine blue of CA1 region of group III (Depressed group) revealed that most of the pyramidal cells had irregular outlines with dark deeply stained nuclei with pericellular spaces (vacuolations) around some of the pyramidal cells. Also, pericellular vacuolation around the glial cells and spacing within the molecular layer and the polymorphic layers were detected, indicating degenerative changes. While examination of the H&E-stained sections and toluidine blue of CA1 region of group IV (Roflumilast-treated group) showed that most of the pyramidal cells had large rounded vesicular nuclei with prominent nucleoli similar of that of the control groups.

Selective GFAP immunohistochemical reaction was used to assess the numerical and structural integrity of hippocampal astrocytes **(60).** Grave neurological conditions as schizophrenia, bipolar disorder, and major depression were included in several scientific studies that proved affection of glial cells structure and function. In the current study an apparent extensive GFAP reaction of the astrocytes with ramification of their processes along the layers of CA1 in group III (Depressed group), controversially, other recent scientific study considered decrease of labeling of astrocytes adjacent to blood vessels as a characteristic feature for schizophrenia and major depression **(61).**

On the other hand, astrocytic GFAP reaction was found to be minimal in CA1 region of the group IV (Roflumilast-treated group) giving apparent similar picture to the group I (Control).

Conclusion:

Therefore, in the current study, it could be concluded that depression is a neurodegenerative inflammatory disease and that oxidative stress plays a key role in depression. This was confirmed through the correlation study which showed that the increased oxidative stress in the depressed rats evidenced by the increased MDA resulted in significant decrease in the CERB, PRKACA and BDNF. On the other side, the increased MDA resulted in significant increase in the PDE4, IL-6.

CERB, PRKACA and BDNF are factors that play a vital role in the neuronal viability and synaptic plasticity in the adult brain that are important in cognitive flexibility and an increased ability to adapt with environmental challenges that may precipitate or exacerbate depressive episodes.

The prolonged oxidative stress led to increased PDE4 which in turn decreased CERB, PRKACA and BDNF and increased IL-6 that the increased neuronal susceptibility to injury and enhanced the development of depression. Besides, it impaired neurogenesis and synaptic plasticity.

In addition, the increased oxidative stress resulted in the decrease of the antioxidant SOD and decrease in the neurotransmitter DA which is normally responsible for the motivation, arousal, reinforcement, and reward encouraging the brain cells to act in a pleasurable, excitable behaviors. This was confirmed by the correlation study that showed decrease in the motor activity in the depressed group e.g. swimming time, struggle time and total no. of crossed squares and the increase in the depressed behavior e.g. significant prolongation in the immobility time, time spent in central zone, latency to leave central zone.

Interestingly in the current study, although corticosteroid level was decreased in the depressed group, there was a significant positive correlation between MDA and corticosteroid level that resulted in altered hippocampal function.

Structural affection was proved histomorphologically in different parts and layers of the hippocampus by depression and was further proved by immunohistochemical reaction (GFAP).

All the previous findings were reversed by Roflumilast treatment (PDE4 inhibitor) which attenuated the depression behavior in rats denoting its neuroprotective, anti-apoptotic, and anti-inflammatory effects.

References

1-Lang, U. E., & Borgwardt, S. (2013). Molecular mechanisms of depression: perspectives on new treatment strategies. *Cellular Physiology and Biochemistry*, *31*(6), 761-777.

2-Boswell, E. B., Anfinson, T. H., & Nemeroff, C. B. (1997). Depression associated with endocrine disorders. *Depression and physical illness*, 255-292.

3-Campbell, S., & MacQueen, G. (2006). An update on regional brain volume differences associated with mood disorders. *Current opinion in psychiatry*, *19*(1), 25-33.

4-Fulton, S., Pissios, P., Manchon, R. P., Stiles, L., Frank, L., Pothos, E. N., ... & Flier, J. S. (2006). Leptin regulation of the mesoaccumbens dopamine pathway. *Neuron*, *51*(6), 811-822.

5-Krügel, U., Schraft, T., Kittner, H., Kiess, W., & Illes, P. (2003). Basal and feeding-evoked dopamine release in the rat nucleus accumbens is depressed by leptin. *European journal of pharmacology*, *482*(1-3), 185-187.

6-Hommel, J. D., Trinko, R., Sears, R. M., Georgescu, D., Liu, Z. W., Gao, X. B., ... & DiLeone, R. J. (2006). Leptin receptor signaling in midbrain dopamine neurons regulates feeding. *neuron*, *51*(6), 801-810.

7-Schellekens, H., Finger, B. C., Dinan, T. G., & Cryan, J. F. (2012). Ghrelin signalling and obesity: at the interface of stress, mood and food reward. *Pharmacology & therapeutics*, *135*(3), 316-326.

8-Dowlati, Y., Herrmann, N., Swardfager, W., Liu, H., Sham, L., Reim, E. K., & Lanctôt, K. L. (2010). A meta-analysis of cytokines in major depression. *Biological psychiatry*, *67*(5), 446-457.

9-Mulinari, S. (2012). Monoamine theories of depression: historical impact on biomedical research. *Journal of the History of the Neurosciences*, *21*(4), 366-392.

10-Gao, F., Yang, S., Wang, J., & Zhu, G. (2022). cAMP-PKA cascade: An outdated topic for depression?. *Biomedicine & Pharmacotherapy, 150*, 113030.

11-Ihara, K., Yoshida, H., Jones, P. B., Hashizume, M., Suzuki, Y., Ishijima, H., ... & Hachisu, M. (2016). Serum BDNF levels before and after the development of mood disorders: a case–control study in a population cohort. *Translational psychiatry*, *6*(4), e782-e782.

12-Gass, P., & Riva, M. A. (2007). CREB, neurogenesis and depression. *Bioessays*, *29*(10), 957-961.

13-Hu, W., Wu, J., Ye, T., Chen, Z., Tao, J., Tong, L., ... & Huang, C. (2020). Farnesoid X receptor-mediated cytoplasmic translocation of CRTC2 disrupts CREB-BDNF signaling in hippocampal CA1 and leads to the development of depression-like behaviors in mice. *International Journal of Neuropsychopharmacology*, *23*(10), 673-686.

14-Zhang, F., Zhu, X., Yu, P., Sheng, T., Wang, Y., & Ye, Y. (2022). Crocin ameliorates depressive-like behaviors induced by chronic restraint stress via the NAMPT-NAD+-SIRT1 pathway in mice. *Neurochemistry International*, *157*, 105343.

15-Li, M. X., Li, Q., Sun, X. J., Luo, C., Li, Y., Wang, Y. N., & Chen, H. (2019). Increased Homer1-mGluR5 mediates chronic stress-induced depressive-like behaviors and glutamatergic dysregulation via activation of PERK-eIF2α. *Progress in Neuro-Psychopharmacology and Biological Psychiatry*, *95*, 109682.

16-Fujimaki, K., Morinobu, S., & Duman, R. S. (2000). Administration of a cAMP phosphodiesterase 4 inhibitor enhances antidepressant-induction of BDNF mRNA in rat hippocampus. *Neuropsychopharmacology*, *22*(1), 42-51.

17-O'Donnell, J. M., & Zhang, H. T. (2004). Antidepressant effects of inhibitors of cAMP phosphodiesterase (PDE4). *Trends in pharmacological sciences*, *25*(3), 158-163.

18- Ashour, N. H., El-Tanbouly, D. M., El Sayed, N. S., & Khattab, M. M. (2021). Roflumilast ameliorates cognitive deficits in a mouse model of amyloidogenesis and tauopathy: Involvement of nitric oxide status, Aβ extrusion transporter ABCB1, and reversal by PKA inhibitor H89. *Progress in Neuro-Psychopharmacology and Biological Psychiatry*, *111*, 110366.

19- Hasan, N., Zameer, S., Najmi, A. K., Parvez, S., Yar, M. S., & Akhtar, M. (2021). Roflumilast and tadalafil improve learning and memory deficits in intracerebroventricular Aβ1–42 rat model

of Alzheimer's disease through modulations of hippocampal cAMP/cGMP/BDNF signaling pathway. *Pharmacological Reports*, *73*, 1287-1302.

20-Wang, H., Zhang, F. F., Xu, Y., Fu, H. R., Wang, X. D., Wang, L., & Zhang, H. T. (2020). The phosphodiesterase-4 inhibitor roflumilast, a potential treatment for the comorbidity of memory loss and depression in Alzheimer's disease: A preclinical study in APP/PS1 transgenic mice. *International Journal of Neuropsychopharmacology*, *23*(10), 700-711.

21-Wu, Q., Qi, L., Li, H., Mao, L., Yang, M., Xie, R., & Sun, B. (2017). Roflumilast reduces cerebral inflammation in a rat model of experimental subarachnoid hemorrhage. *Inflammation*, *40*, 1245-1253.

22-Bravo, J. A., Díaz-Veliz, G., Mora, S., Ulloa, J. L., Berthoud, V. M., Morales, P., & Fiedler, J. L. (2009). Desipramine prevents stress-induced changes in depressive-like behavior and hippocampal markers of neuroprotection. *Behavioural pharmacology*, *20*(3), 273-285.

23-Tõnissaar, M., Mällo, T., Eller, M., Häidkind, R., Kõiv, K., & Harro, J. (2008). Rat behavior after chronic variable stress and partial lesioning of 5-HT-ergic neurotransmission: effects of citalopram. *Progress in Neuro-Psychopharmacology and Biological Psychiatry*, *32*(1), 164-177.

24-Flecknell, P., Lofgren, J. L., Dyson, M. C., Marini, R. R., Swindle, M. M., & Wilson, R. P. (2015). Preanesthesia, anesthesia, analgesia, and euthanasia. In *Laboratory animal medicine* (pp. 1135-1200). Academic Press.

25-Scudamore, C. L. (2014). *A Practical Guide to the Histology of the Mouse*. John Wiley & Sons.

26-Bradford, M. M. (1976). A rapid and sensitive method for the quantitation of microgram quantities of protein utilizing the principle of protein-dye binding. *Analytical biochemistry*, *72*(1-2), 248-254.

27-Bancroft, J. and Gamble, M. 2002. Theory and Practice of Histological Techniques. 5th ed., Churchill Livingstone publishers, Edinburgh, UK.

28-Graham, L. and Orenstein, J.M. Processing tissue and cells for transmission electron microscopy in diagnostic pathology and research. Nature Proto-cols 2007 ;2 (10): 2439-2450.

29-Amal, A. M. (2014). Possible protective effect of Ascorbic acid and Alpha-Tocopherol Against damage induced by ionizing radiation in adult albino rat hippocampus. Egyptian journal of Anatomy, 37(2): 303-323.

30-Strekalova, T., Spanagel, R., Bartsch, D., Henn, F. A., & Gass, P. (2004). Stress-induced anhedonia in mice is associated with deficits in forced swimming and exploration. *Neuropsychopharmacology*, *29*(11), 2007-2017.

31-Aboul-Fotouh, S. (2013). Coenzyme Q10 displays antidepressant-like activity with reduction of hippocampal oxidative/nitrosative DNA damage in chronically stressed rats. *Pharmacology Biochemistry and Behavior*, *104*, 105-112.

32- Feng H, Wang C, He W, Wu X, Li S, Zeng Z, Wei M, He B (2019) Roflumilast ameliorates cognitive impairment in APP/PS1 mice via cAMP/CREB/BDNF signaling and anti-neuroinflammatory effects. *Metab Brain Dis* 34:583–591.

33-Pushpa, V.H., Jayanthi, M.K. and Ramu, R (2021). EVALUATION OF ANTI-DEPRESSANT ACTIVITY OF ROFLUMILAST IN ALBINO MICE. International Journal of Pharmaceutical Sciences and Research,; 12(9): 5051-5056.

34-Hasan, N., Zameer, S., Najmi, A. K., Parvez, S., & Akhtar, M. (2022). Roflumilast Reduces Pathological Symptoms of Sporadic Alzheimer's Disease in Rats Produced by Intracerebroventricular Streptozotocin by Inhibiting NF-κB/BACE-1 Mediated Aβ Production in

the Hippocampus and Activating the cAMP/BDNF Signalling Pathway. *Neurotoxicity Research*, *40*(2), 432-448.

35-Waltrick, A. P. F., da Silva, A. C. F., de Mattos, B. A., Chaves, Y. C., de Oliveira, R. M. W., Prickaerts, J., & Zanoveli, J. M. (2023). Long-term treatment with roflumilast improves learning of fear extinction memory and anxiety-like response in a type-1 diabetes mellitus animal model. *Behavioural Brain Research*, *439*, 114217.

36-Pivonello, R., Isidori, A. M., De Martino, M. C., Newell-Price, J., Biller, B. M., & Colao, A. (2016). Complications of Cushing's syndrome: state of the art. *The lancet Diabetes & endocrinology*, *4*(7), 611-629.

37-Feng, X., Zhao, Y., Yang, T., Song, M., Wang, C., Yao, Y., & Fan, H. (2019). Glucocorticoid-driven NLRP3 inflammasome activation in hippocampal microglia mediates chronic stress-induced depressive-like behaviors. *Frontiers in molecular neuroscience*, *12*, 210.

38-Sanders, O., & Rajagopal, L. (2020). Phosphodiesterase inhibitors for Alzheimer’s disease: a systematic review of clinical trials and epidemiology with a mechanistic rationale. *Journal of Alzheimer's Disease Reports*, *4*(1), 185-215.

39-Ruan, J., Liu, L., Shan, X., Xia, B., & Fu, Q. (2019). Anti-depressant effects of oil from fructus gardeniae via PKA-CREB-BDNF signaling. *Bioscience Reports*, *39*(4)

40-Essam, R. M., Ahmed, L. A., Abdelsalam, R. M., & El-Khatib, A. S. (2019). Phosphodiestrase-1 and 4 inhibitors ameliorate liver fibrosis in rats: Modulation of cAMP/CREB/TLR4 inflammatory and fibrogenic pathways. *Life sciences*, *222*, 245-254

41-Zhong, F., Liu, L., Wei, J. L., Hu, Z. L., Li, L., Wang, S., ... & Dai, R. P. (2019). Brain-derived neurotrophic factor precursor in the hippocampus regulates both depressive and anxiety-like behaviors in rats. *Frontiers in Psychiatry*, *9*, 776.

42-Duman, R. S., & Monteggia, L. M. (2006). A neurotrophic model for stress-related mood disorders. *Biological psychiatry*, *59*(12), 1116-1127.

43-Brunoni, A. R., Lopes, M., & Fregni, F. (2008). A systematic review and meta-analysis of clinical studies on major depression and BDNF levels: implications for the role of neuroplasticity in depression. *The The International Journal of Neuropsychopharmacology*, *11*(8), 1169-1180.

44-Zhang, C., Xing, Z., Tan, M., Wu, Y., & Zeng, W. (2021). Roflumilast ameliorates isoflurane-induced inflammation in astrocytes via the CREB/BDNF Signaling Pathway. *ACS omega*, *6*(6), 4167-4174.

45-Gong, H., Su, W. J., Cao, Z. Y., Lian, Y. J., Peng, W., Liu, Y. Z., ... & Jiang, C. L. (2018). Hippocampal Mrp8/14 signaling plays a critical role in the manifestation of depressive-like behaviors in mice. *Journal of neuroinflammation*, *15*(1), 1-13.

46-Jeon, S. W., & Kim, Y. K. (2016). Neuroinflammation and cytokine abnormality in major depression: Cause or consequence in that illness?. *World journal of psychiatry*, *6*(3), 283.

47-Kim, Y. K., Na, K. S., Myint, A. M., & Leonard, B. E. (2016). The role of pro-inflammatory cytokines in neuroinflammation, neurogenesis and the neuroendocrine system in major depression. *Progress in Neuro-Psychopharmacology and Biological Psychiatry*, *64*, 277-284.

48-Moreines, J. L., Owrutsky, Z. L., Gagnon, K. G., & Grace, A. A. (2017). Divergent effects of acute and repeated quetiapine treatment on dopamine neuron activity in normal vs. chronic mild stress induced hypodopaminergic states. *Translational Psychiatry*, *7*(12), 1275.

49-Savitz, J. B., & Drevets, W. C. (2013). Neuroreceptor imaging in depression. *Neurobiology of disease*, *52*, 49-65.

50Gilleen, J., Nottage, J., Yakub, F., Kerins, S., Valdearenas, L., Uz, T., ... & Shergill, S. S. (2021). The effects of roflumilast, a phosphodiesterase type-4 inhibitor, on EEG biomarkers in schizophrenia: A randomised controlled trial. *Journal of Psychopharmacology*, *35*(1), 15-22.

51-Dremencov, E., Grinchii, D., Hrivikova, K., Lapshin, M., Komelkova, M., Graban, J., ... & Jezova, D. (2022). Exposure to chronic stressor upsurges the excitability of serotoninergic neurons and diminishes concentrations of circulating corticosteroids in rats two weeks thereafter. *Pharmacological Reports*, *74*(3), 451-460.

52-Dröge, W. (2002). Free radicals in the physiological control of cell function. *Physiological reviews*.

53-Bajpai, A., Verma, A. K., Srivastava, M., & Srivastava, R. (2014). Oxidative stress and major depression. *Journal of clinical and diagnostic research: JCDR*, *8*(12), CC04.

54-Bhatt, S., Nagappa, A. N., & Patil, C. R. (2020). Role of oxidative stress in depression. *Drug discovery today*, *25*(7), 1270-1276.

55-Tuon, T., Meirelles, S. S., de Moura, A. B., Rosa, T., Borba, L. A., Botelho, M. E. M., ... & Reus, G. Z. (2021). Behavior and oxidative stress parameters in rats subjected to the animal's models induced by chronic mild stress and 6-hydroxydopamine. *Behavioural Brain Research*, *406*, 113226.

56-Czeh B, Varga ZK, Henningsen K, Kovacs GL, Miseta A, Wiborg O. 2015. Chronic stress reduces the number of GABAergic interneurons in the adult rat hippocampus, dorsal-ventral and regionspecific differences. Hippocampus 25:393–405.

57-Godavarthi SK, Sharma A, Jana NR. 2014. Reversal of reduced parvalbumin neurons in hippocampus and amygdala of Angelman syndrome model mice by chronic treatment of fluoxetine. J Neurochem 130:444–454.

58-Megahed T, Hattiangady B, Shuai B, Shetty AK. 2015. Parvalbumin and neuropeptide Y expressing hippocampal GABA-ergic inhibitory interneuron numbers decline in a model of Gulf War illness. Front Cell Neurosci 8:447.

59-Milner TA, Burstein SR, Marrone GF, Khalid S, Gonzalez AD, Williams TJ, Schierberl KC, Torres-Reveron A, Gonzales KL, McEwen BS, Waters EM. 2013. Stress differentially alters mu opioid receptor density and trafficking in parvalbumin-containing interneurons in the female and male rat hippocampus. Synapse 67:757–772.

60-Webster MJ, Knable MB, Johnston-Wilson N, Nagata K, Inagaki M, Yolken RH (2001) Immunohistochemical localization of phosphorylated glial fibrillary acidic protein in the prefrontal cortex and hippocampus from patients with schizophrenia, bipolar disorder, and depression. Brain Behav Immun 15:388–400

61-Ayuob, N. N., Firgany, A. E. D. L., El-Mansy, A. A., & Ali, S. (2017). Can Ocimum basilicum relieve chronic unpredictable mild stress-induced depression in mice?. *Experimental and molecular pathology*, *103*(2), 153-161.

Figure Click here to access/download;Figure;Biochemical Figures.docx

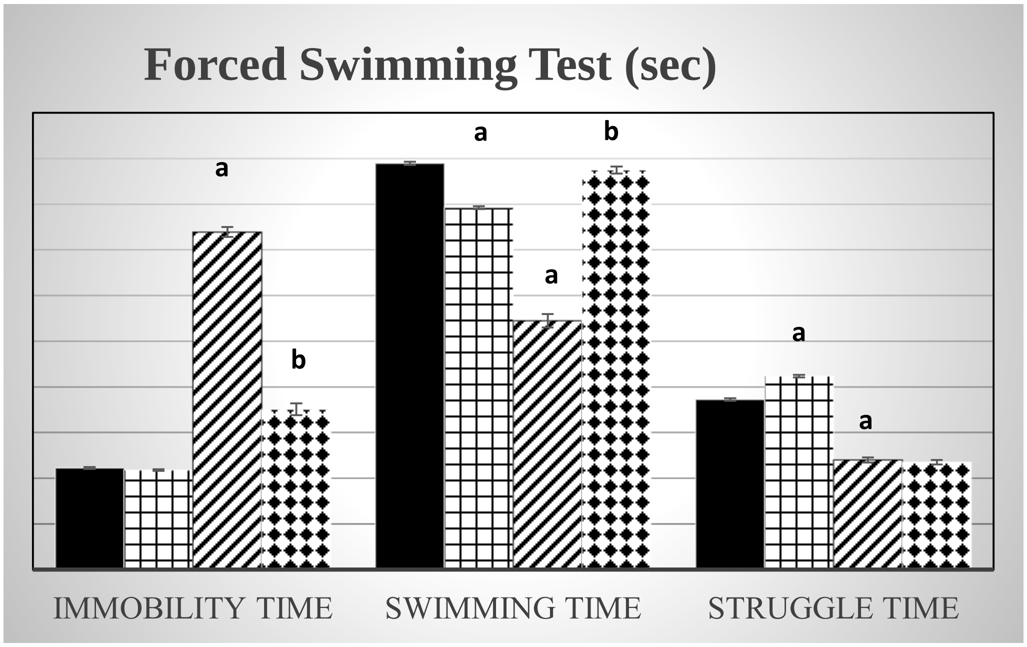

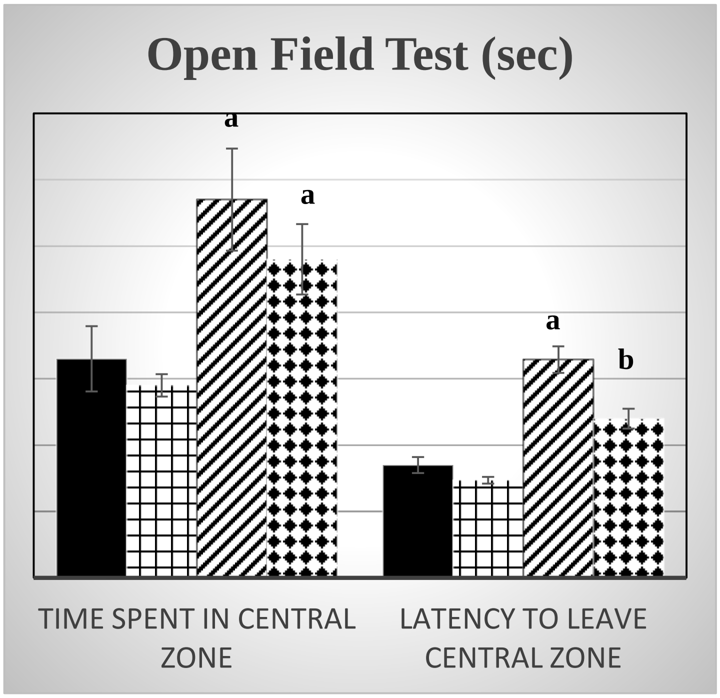

Figure (1): Behavioral tests changes in in the different studied groups.
a: Significance by LSD at P<0.05 from control group
b: Significance by LSD at P<0.05 from depressed group

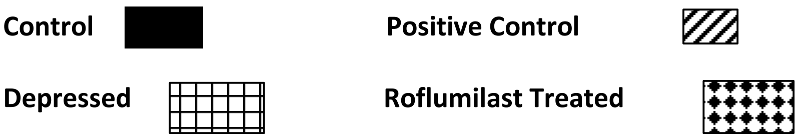

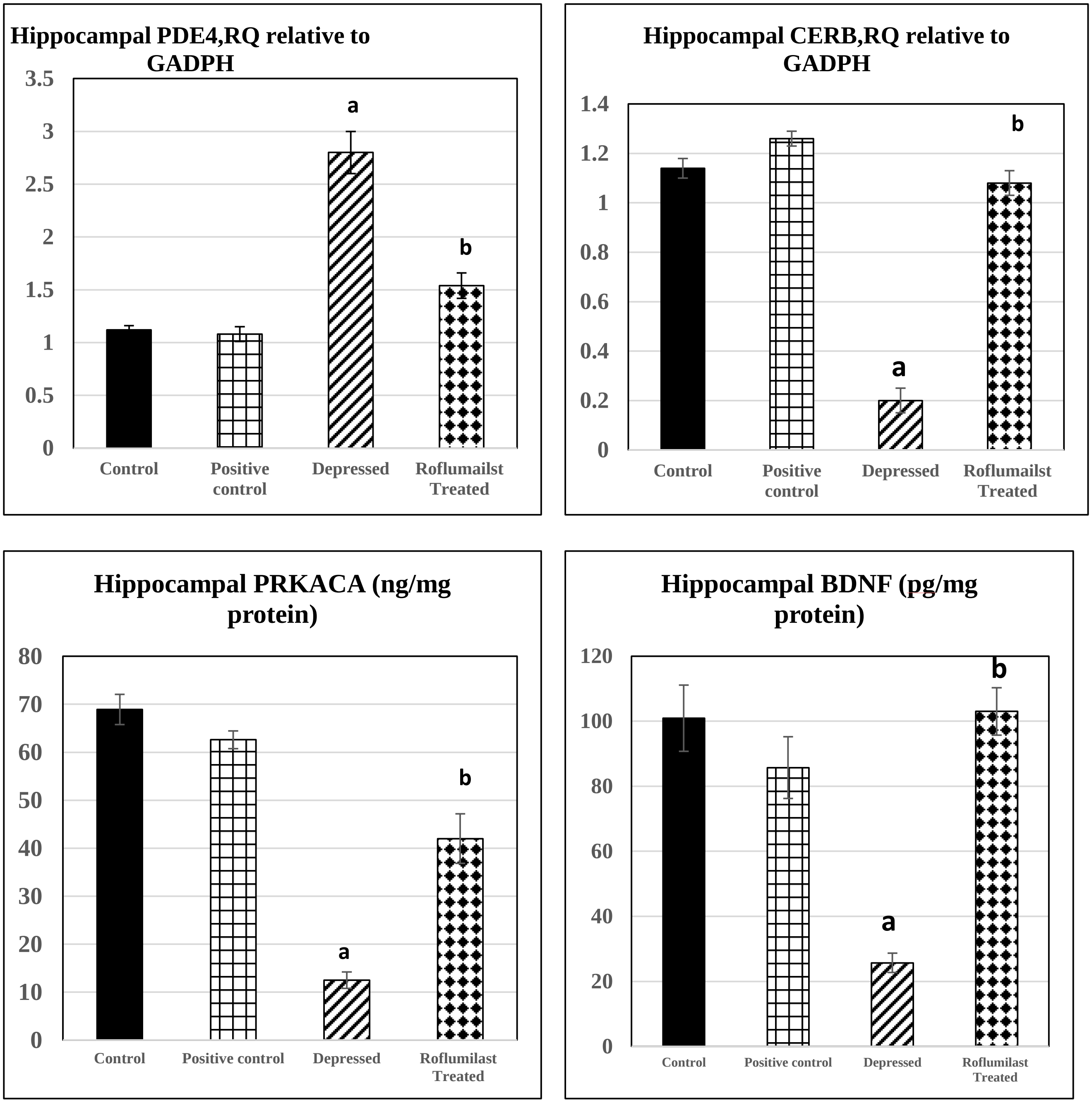

Figure (2): Changes in Hippocampal levels of phosphodiesterase 4, CERB, PKA and BDNF in the different studied groups.

a: Significance by LSD at P<0.05 from control group

b: Significance by LSD at P<0.05 from depressed group

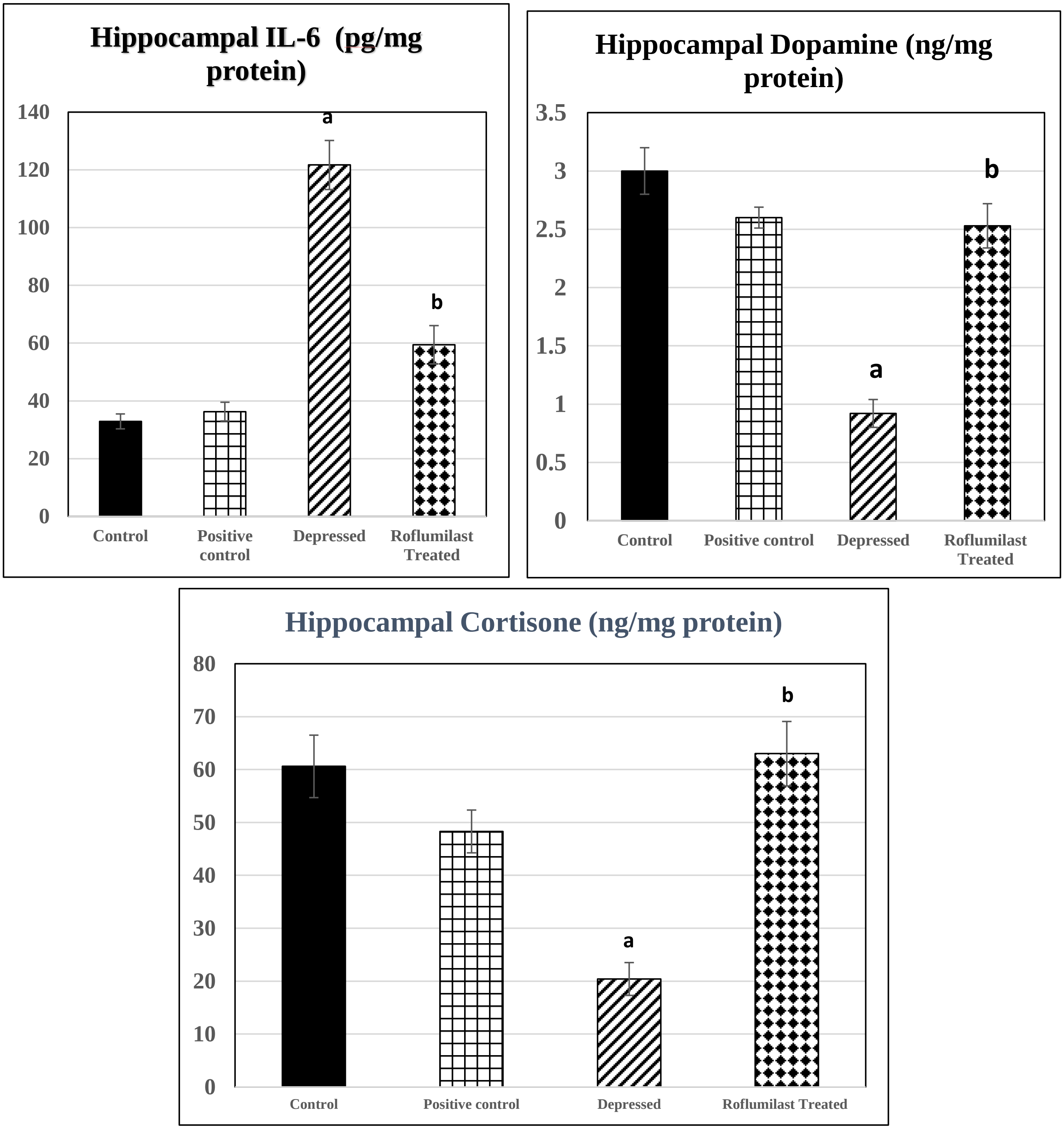

Figure (3): Changes in Hippocampal levels of IL6, dopamine and cortisone in the different studied groups.

a: Significance by LSD at P<0.05 from control group

b: Significance by LSD at P<0.05 from depressed group

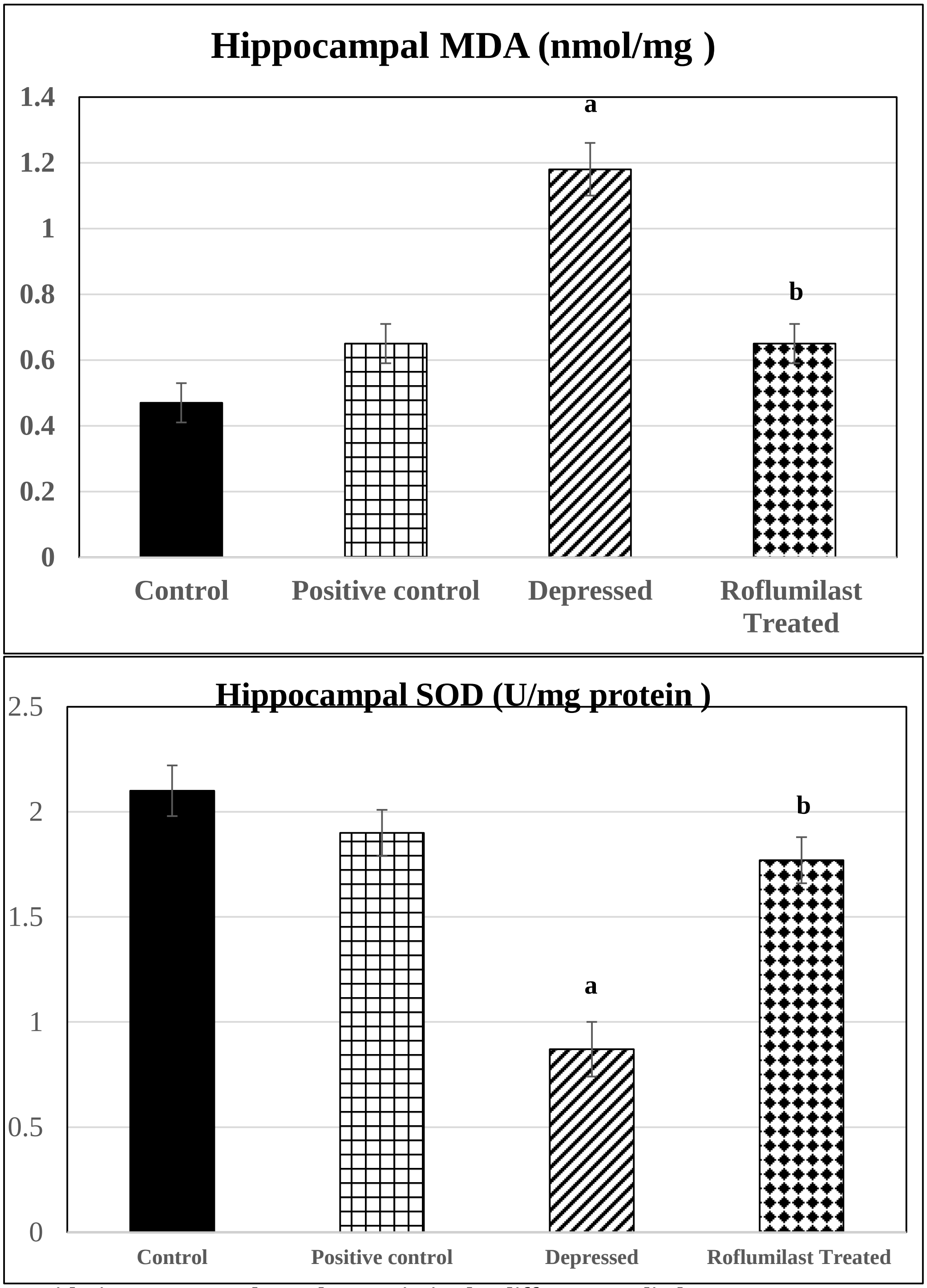

Figure (4): Oxidative stress markers changes in in the different studied groups.

a: Significance by LSD at P<0.05 from control group

b: Significance by LSD at P<0.05 from depressed group

Figure

Click here to access/download;Figure;Histology figures.pdf

Fig. 5

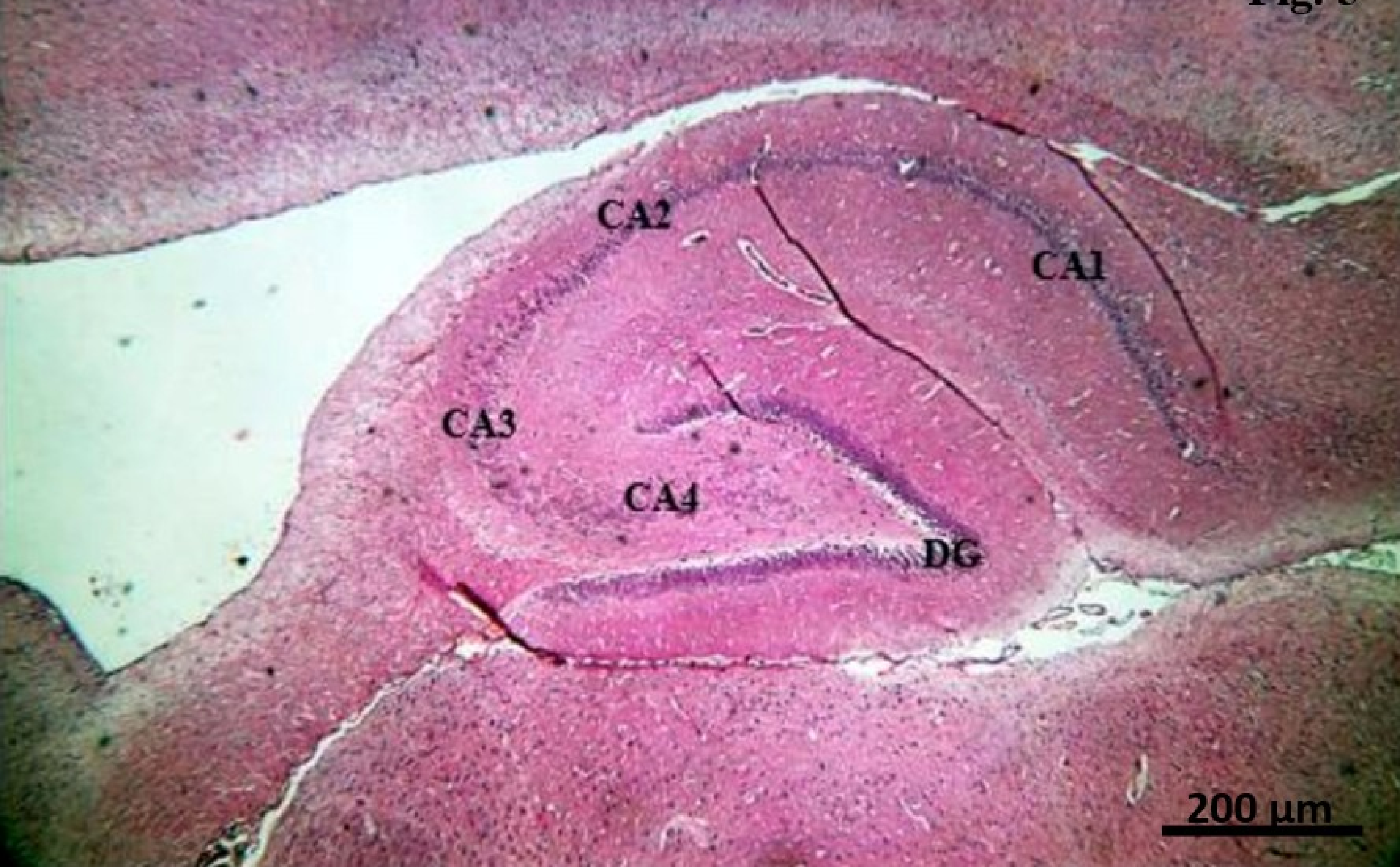

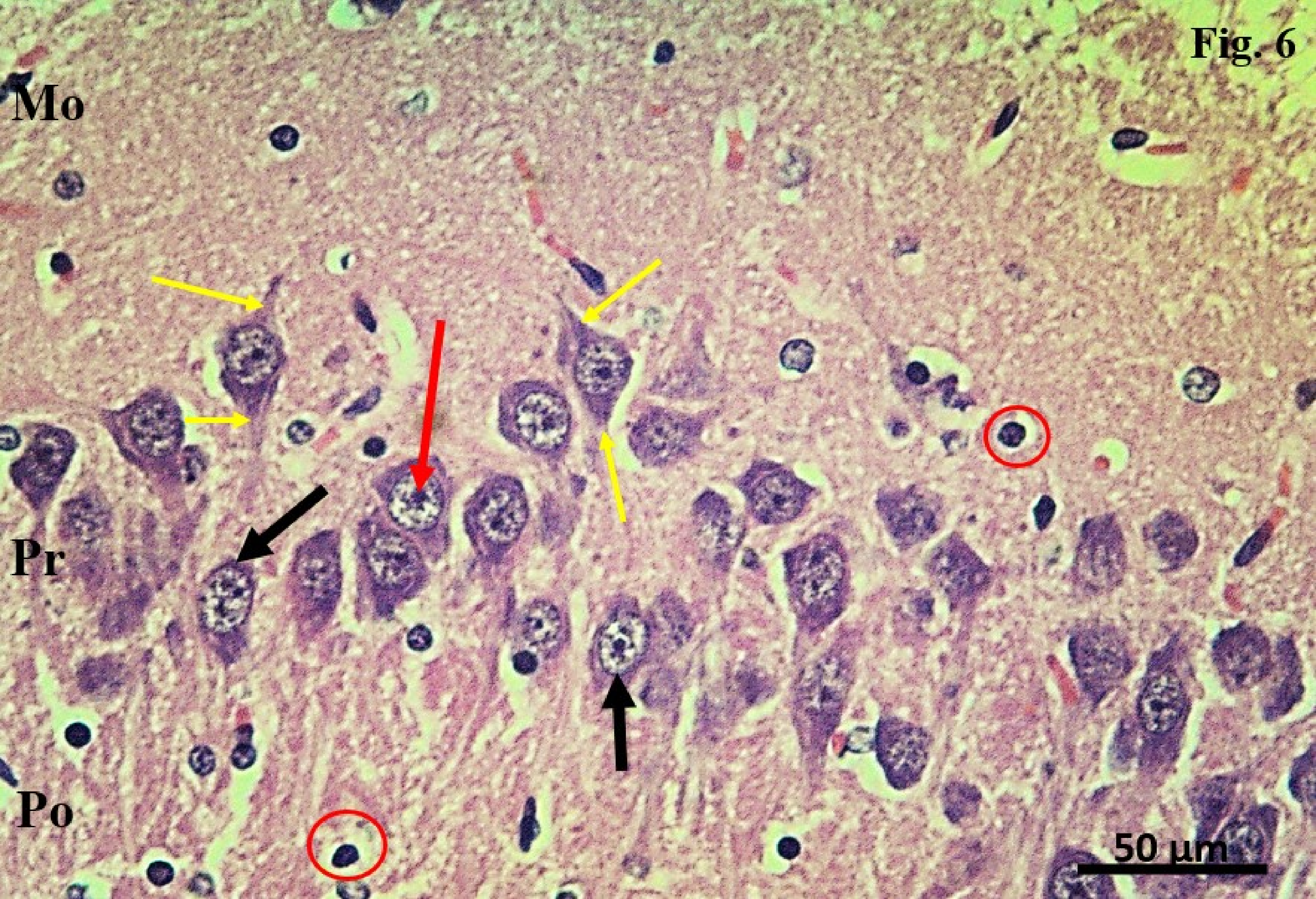
Fig. 6
Mo
Pr
Po
50 µm

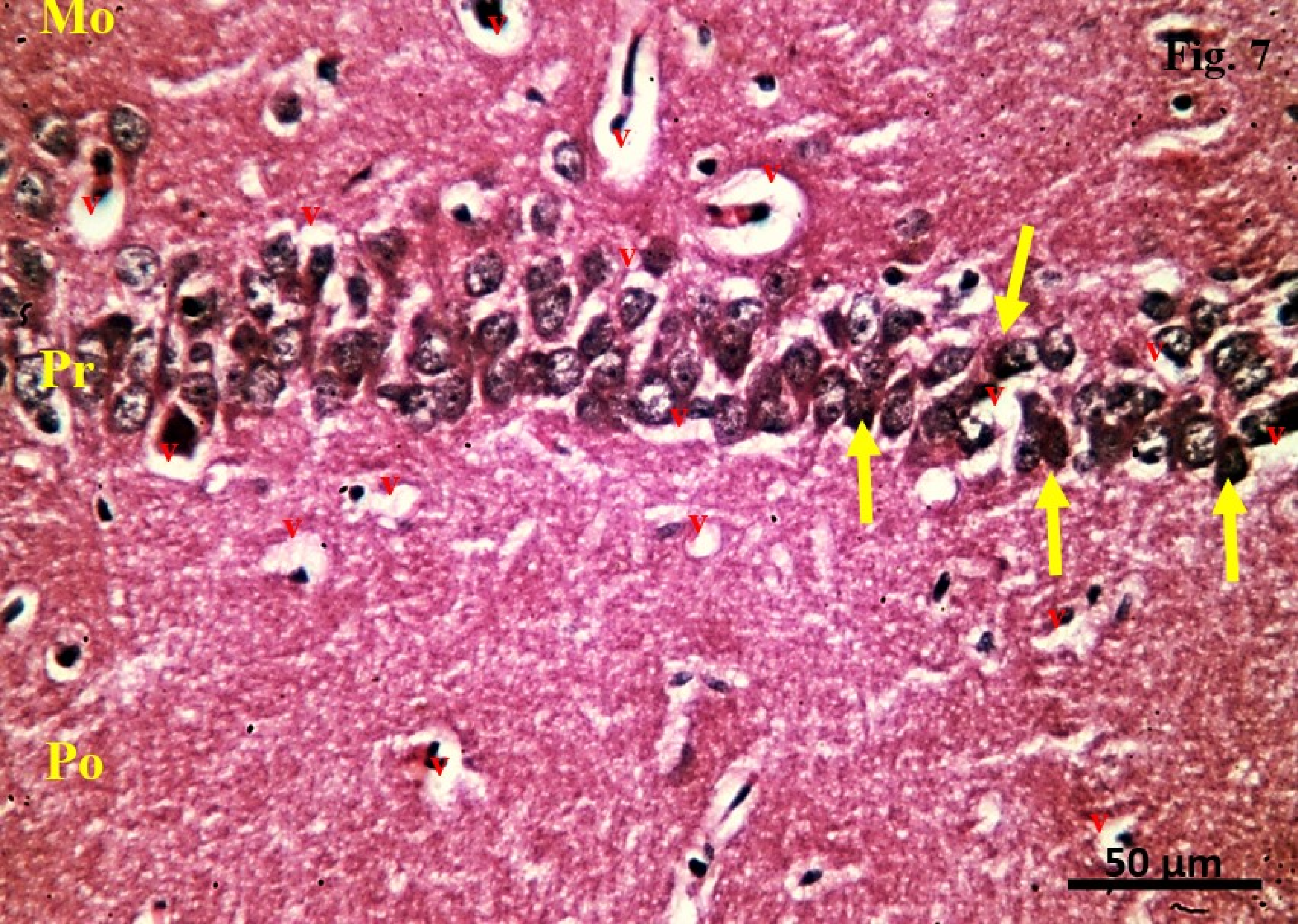
Fig. 7
Mo
Pr
Po
50 µm

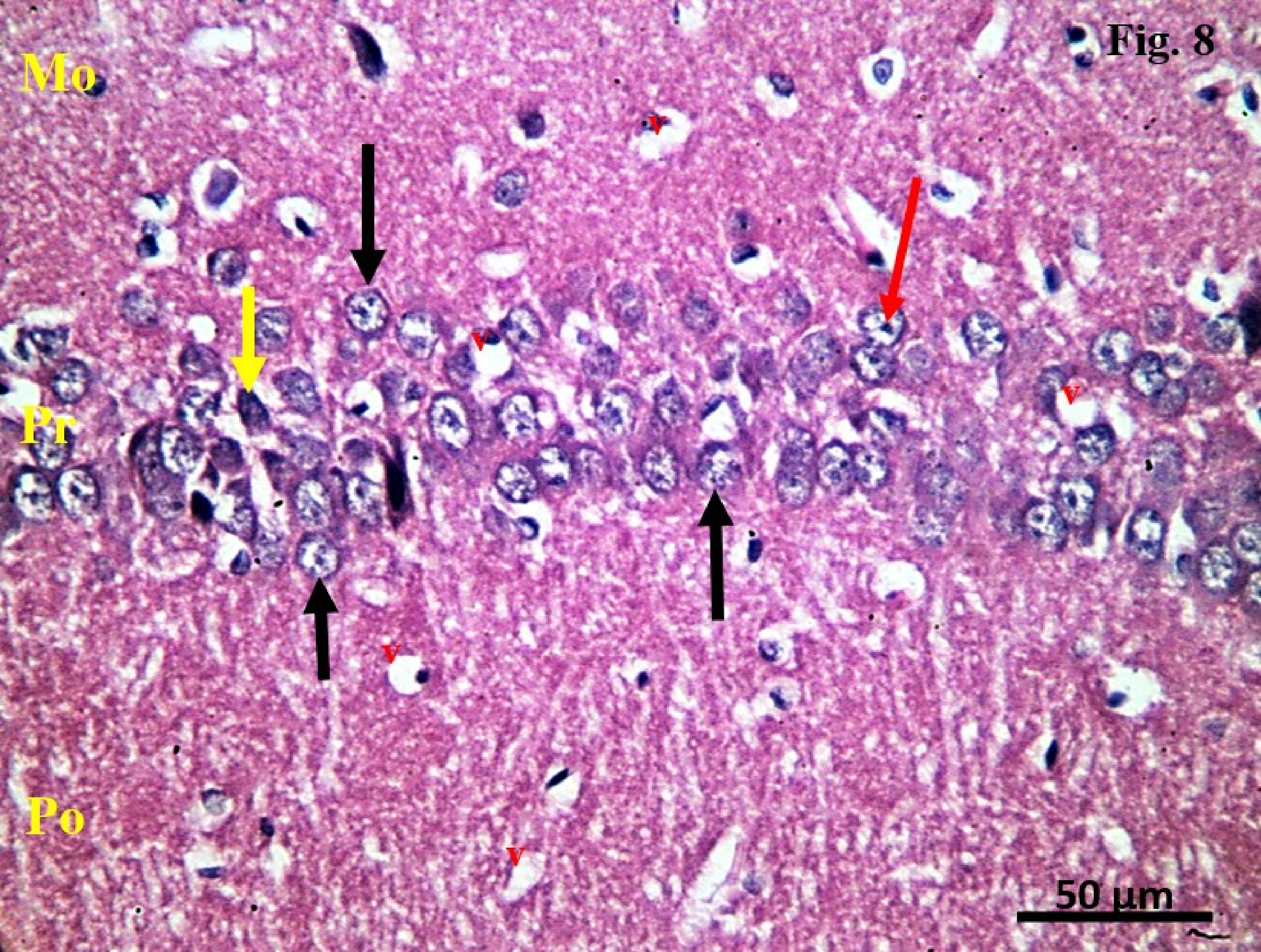
Fig. 8
Mo
Pr
Po
v
50 µm

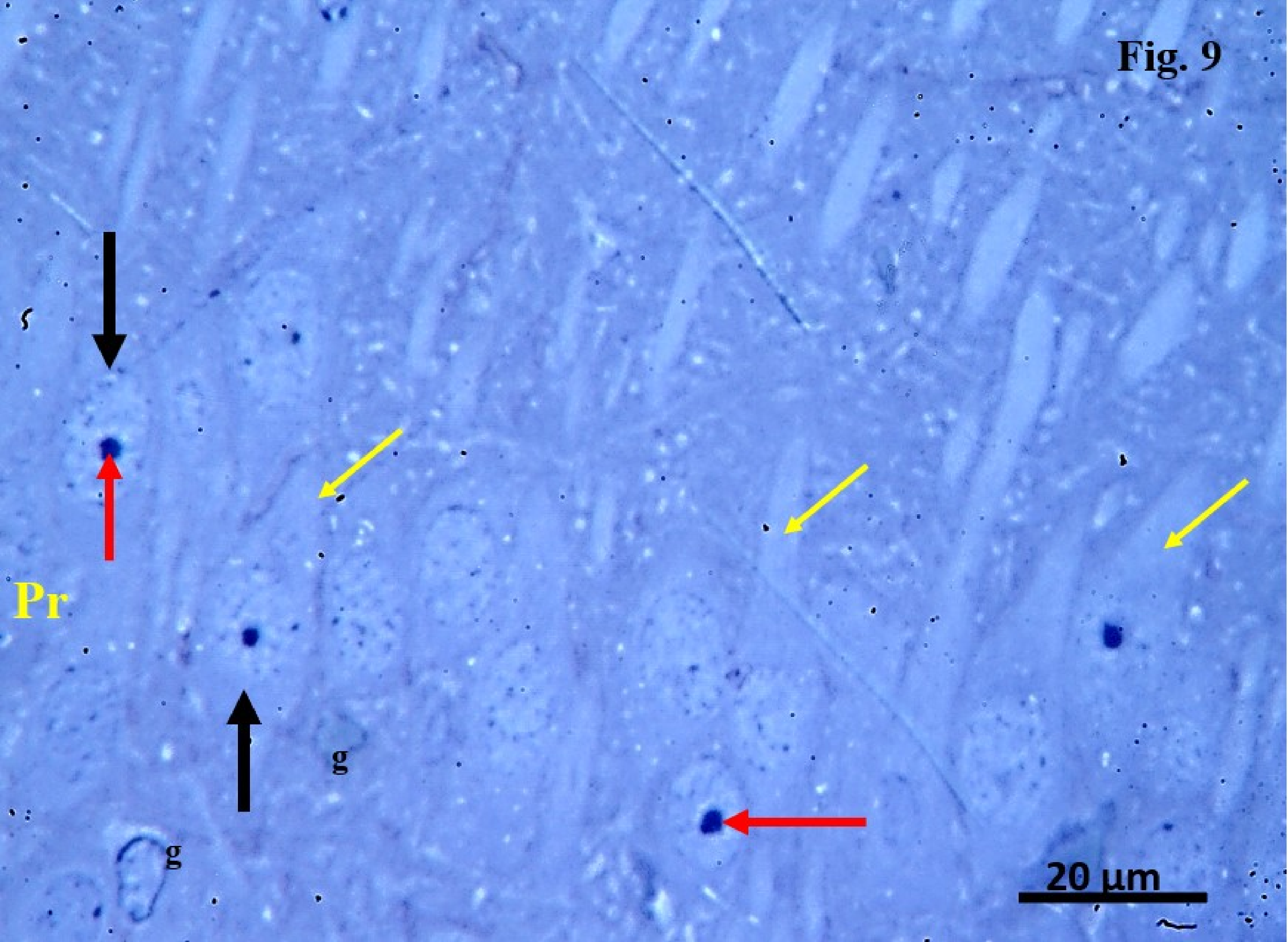
Fig. 9
Pr
g
g
20 µm

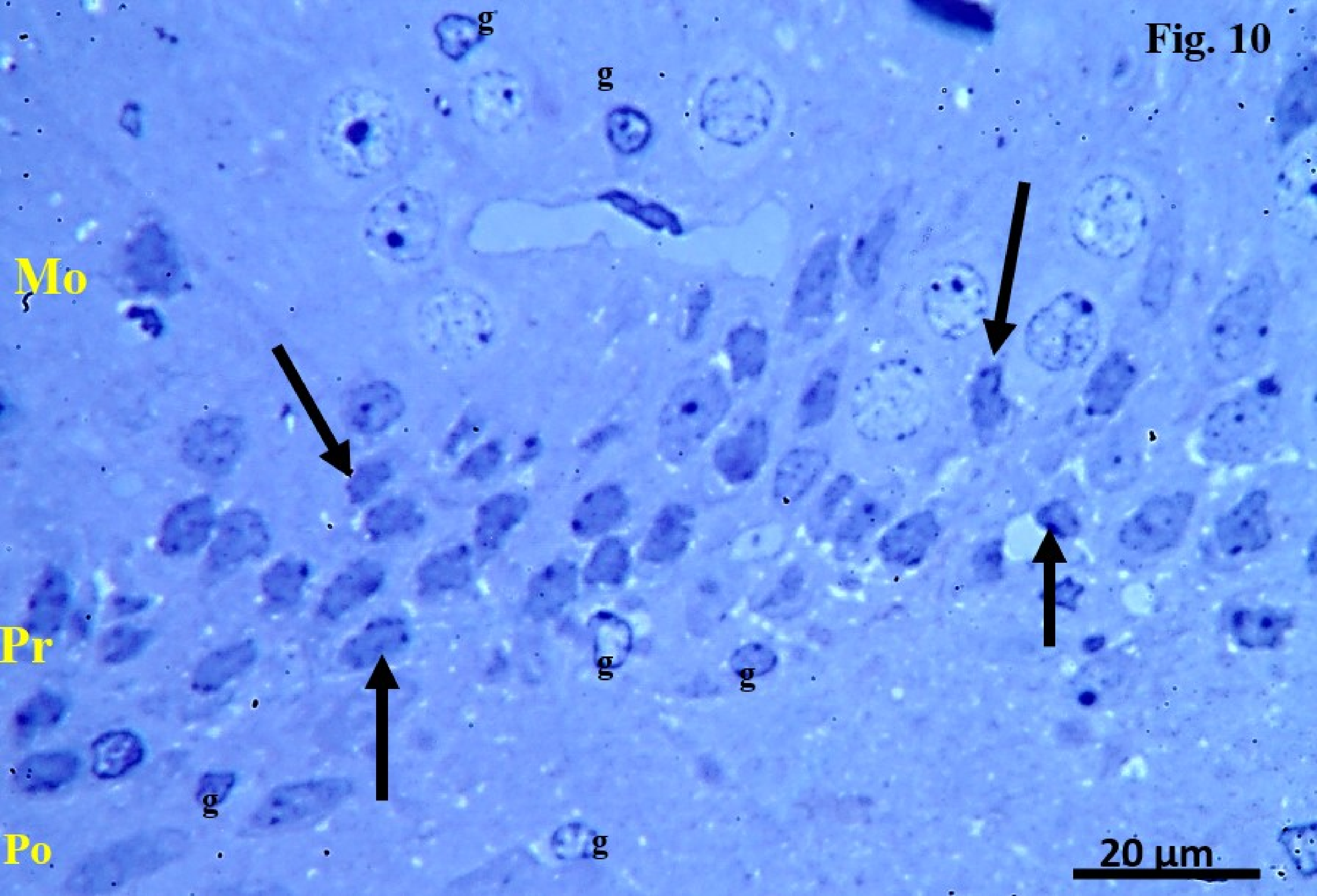
Fig. 10
Mo
Pr
Po
g
g
g
g
g
g
20 µm

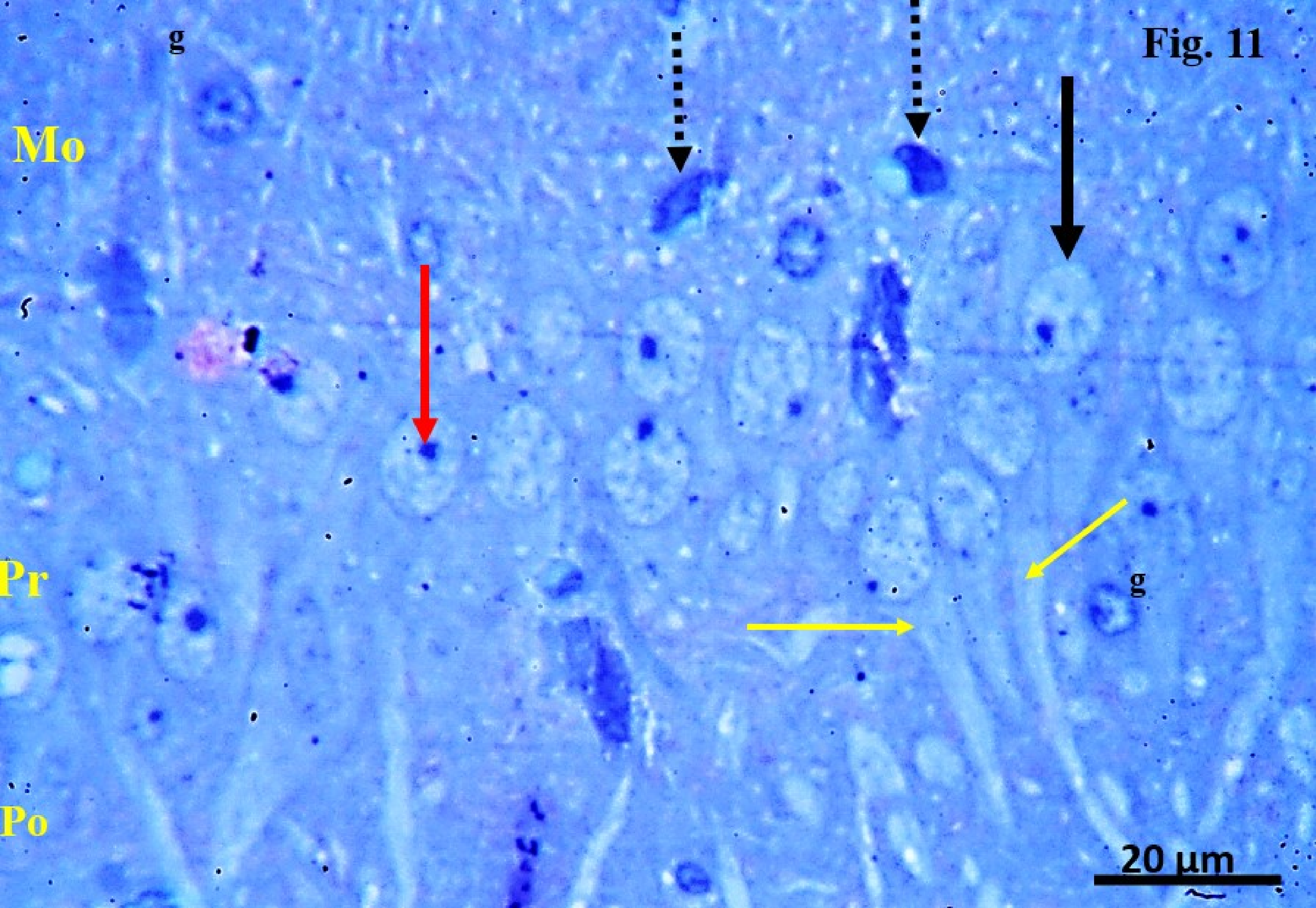
g
Fig. 11
Mo
Pr
g
Po
20 µm

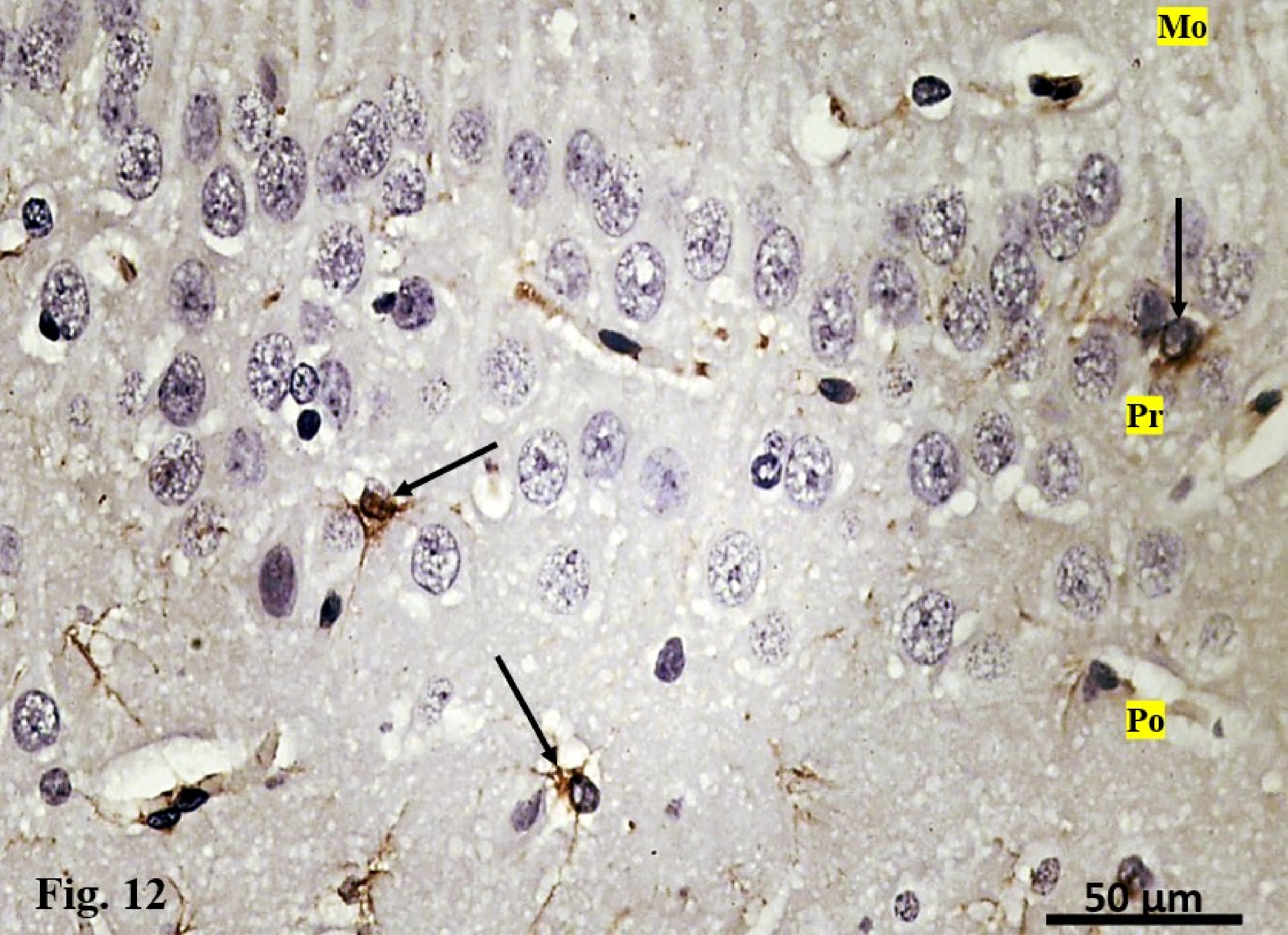

Mo
Pr
Po
Fig. 12
50 µm

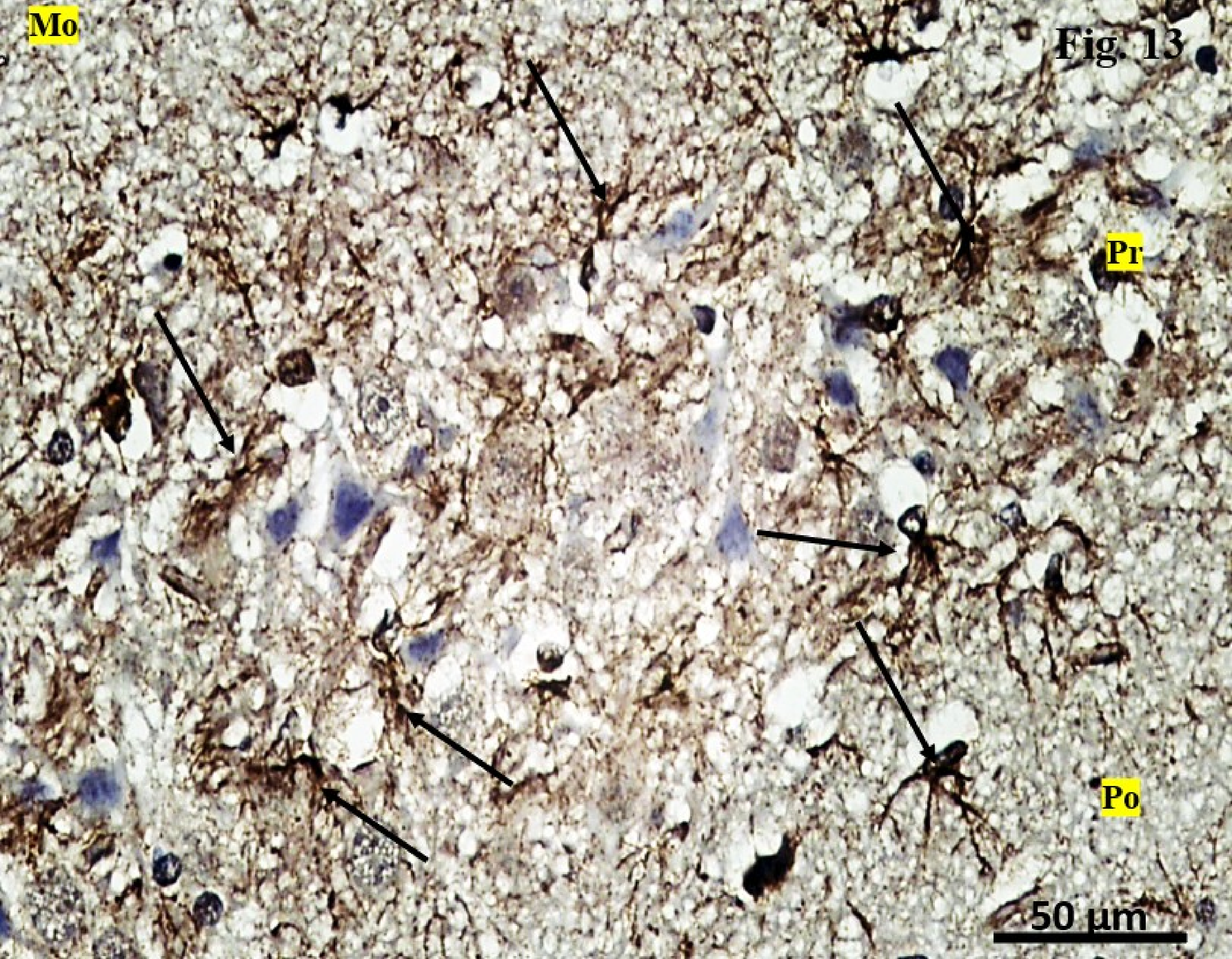
Mo
Fig. 13
Pr
Po
50 µm

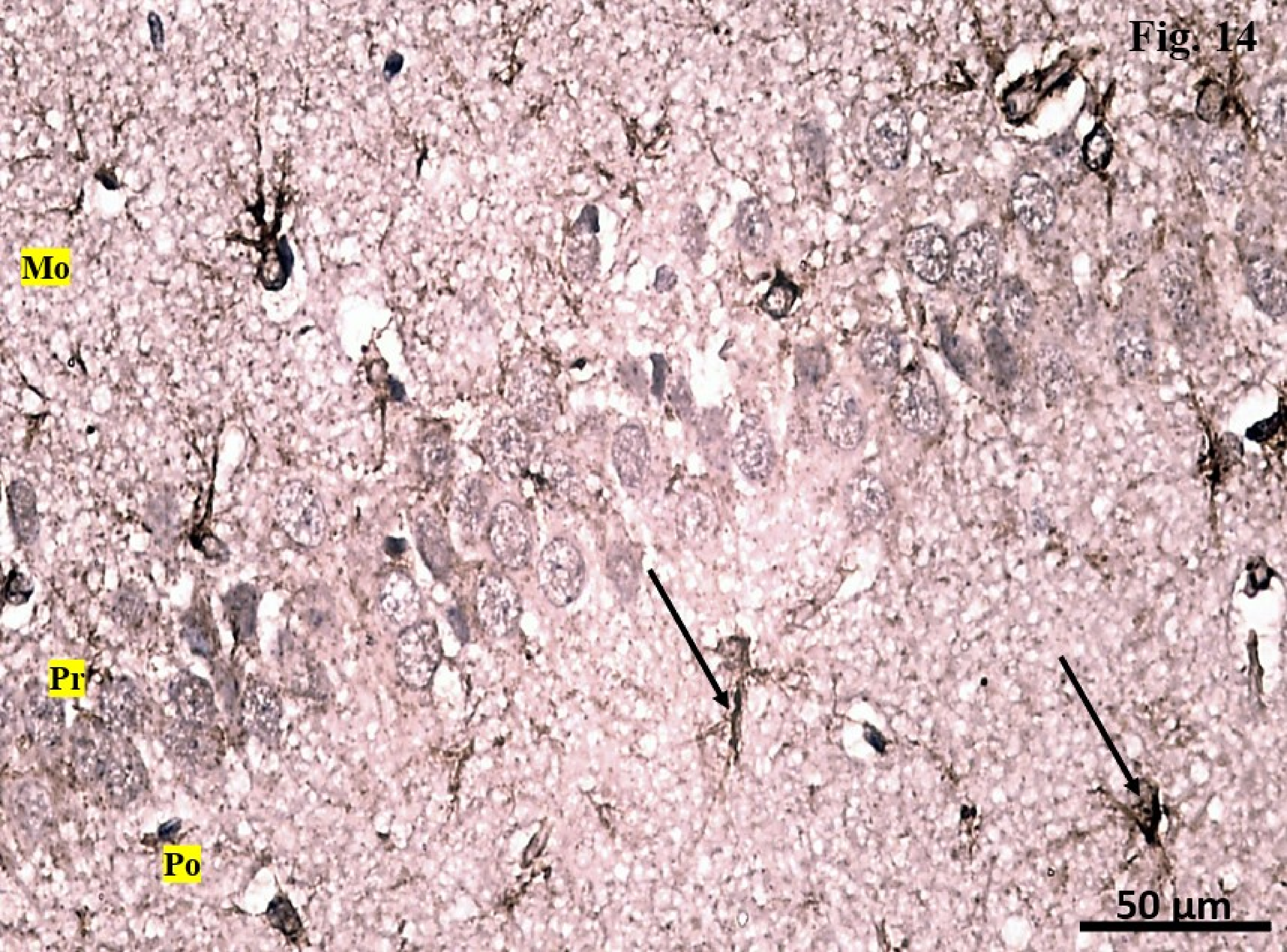
Fig. 14
Mo
Pr
Po
50 µm
