# Supplementary material for: A study of roflumilast treatment on functional and structural changes in hippocampus in depressed Adult male Wistar rats
Source: PLoS One. 2024 Feb 5;19(2):e0296187. doi: 10.1371/journal.pone.0296187 (PMC10843119; doi:10.1371/journal.pone.0296187)
Supplement: Attachment — Submitted filename: Comments.docx [file pone.0296187.s001.docx]

**Study of Roflumilast Treatment on Functional and Structural Changes in Hippocampus in Depressed Adult Male Wistar Rats**

**Comments**

The idea of the article is interesting, and the manuscript is well written. The experiments are described in sufficient detail. However, a major revision is recommended. The following points need to be addressed:

1. The antidepressant effects of the PDEIs are reported in several studies. Why roflumilast was specifically chosen in this study?

2. The authors cited a recent study (Wang et al. (2020), (20)) which reported that “roflumilast not only improves learning and memory but also attenuates depression-like behavior in AD mice, likely via PDE4B/PDE4D-mediated cAMP/cAMP response element-binding protein/brain derived neurotrophic factor signaling”.

What are the different findings between this study and your study?

3. No need for these words “Aim of the work”

4. Kindly include in Materials and methods the information about justification for use of animals, housing, feeding, and environmental enrichment, and the steps taken to minimize suffering of animals.

5. The authors mentioned that (Rats were randomly divided into four groups: -Control group (n=10); which…..- Positive control group (n=10); which…- Depressed group (n=10),…. - Roflumilast-treated group (n=10):….). Please rewrite as follows: Rats were randomly divided into four groups: (1) Control group (n=10); which…… (2) Positive control group (n=10); which…(3) Depressed group (n=10),…. (4) Roflumilast-treated group (n=10):….

6. The name of the supplier of roflumilast should be added.

7. The authors mentioned that “Roflumilast-treated group (n=10): rats were exposed to chronic restraint stress 4 hours per day for 6 weeks and were treated by Roflumilast (WESTABREATH 500 ®) 3 mg / kg / day, by oral

gavage, dissolved in distilled water for the last 4 weeks of the experiment”. This indicates that roflumilast may just interfere with full development of the model i.e., prophylactic effect. Why didn’t the authors try a therapeutic protocol?

8. Preparation of tissue homogenate: …according to manual instructions…. Please add ‘the’ before manual and add a reference.

9. Determination of tissue protein: the catalog numbers of the kits are missing.

10. No need for this repetition: (P≤0.001, P≤0.001, P≤0.001 respectively), just mention P≤0.001 for all. Please correct accordingly in all results.

11. The legends for figures 2,3, and 4 need to be adjusted and the key for the different groups must be added in the figure itself, not in its legend. For example, change legend of Figure (1) as follows: Effects of Roflumilast on: (A) Forced swimming test and (B) Open Field test in Depressed Adult Male Wistar Rats (n=10). a: P<0.05 *versus* control group, b: P<0.05 *versus* depressed group. It’s better to use more letters to add the exact P values.

12. For figures of H&E-stained sections (Figs 5-8), the scale bars must be the same. Also, collect them in one figure as one plate for better clarity and easier comparison.

13. Toluidine blue stained semithin sections, and Immunohistochemical (GFAP) stained sections: where are the images of the positive control? Please collect all sections having the same stain in one figure as one plate for better clarity and easier comparison.

14. Table (1): correlations of MDA and other parameters in all studied groups. Please correct to Table (1): Correlations between MDA and other parameters in all studied groups. And explain what abbreviations mean. Apply the same for tables 2 and 3.

15. What do you mean by this legend? Fig (2) & Fig (3): Changes in Hippocampal levels of phosphodiesterase 4 and IL6 in the different studied groups.

16. The authors mentioned: (Similarly, Wang et al. (2020) found decreased immobility time and)… The number of the reference (20) is missing.

17. There are some typos like: …neurotropshic factors in many glucocorticoid receptor…Please correct to neurotrophic. Please check the whole manuscript.

18. In conclusion: please delete (Therefore, in the current study, it could be concluded that)

19. Discussion and conclusion sections are very long, please shorten both.

20. Please add a paragraph to explain the limitations of the study
