# Supplementary material for: A study of roflumilast treatment on functional and structural changes in hippocampus in depressed Adult male Wistar rats
Source: PLoS One. 2024 Feb 5;19(2):e0296187. doi: 10.1371/journal.pone.0296187 (PMC10843119; doi:10.1371/journal.pone.0296187)
Supplement: Attachment — Submitted filename: Response to Reviewer-2 word.docx [file pone.0296187.s003.docx]

Editor-in-Chief,

**Plos One.**

Thank you very much for reviewing our manuscript. We greatly appreciate the reviewer for his complimentary comments and suggestions. We have revised the manuscript according to the reviewers' suggestion, Please find a point-by-point response to reviewer’s concerns. We hope that you find our responses satisfactory and that the manuscript is now acceptable for publication.

Sincerely,

Ghida Hassan

**The followings are our point-by-point responses to Comments by the reviewers**

**1-Page 8: The antiapoptotic not measured in this study**

**Response**:

Antiapoptotic is removed in the revised manuscript

**2-Page 12: Suppose received drug for depression treatment.**

**Response**:

Stress has increasing incidence nowadays due to exposure to various daily events, so our study was designed to reduce stress within its occurrence which may mimic what happened to humans in real life. We assumed that the patient would take the medication while he is exposed to the same stressors.

**3- Page 14: from which hemisphere, RNA was extracted?**

**Response**:

The study was on the hippocampus. Because hippocampul size was small, we extracted the whole hippocampus from both hemispheres

**4- Page 15: from which hemisphere, brain tissuewas taken for this procedure?**

**Response**:

The study was on the hippocampus. Because hippocampul size was small, we extracted the whole hippocampus from both hemispheres

**5- Page 16: which hemisphere?**

**Response**:

The study was on the hippocampus. Because hippocampul size was small, we extracted the whole hippocampus from both hemispheres

**6- Page 17: Why this happen? Any explanation about this.**

**Response**:

Although roflumilast caused non-significant change of biochemical markers in positive control rats, it may cause some behavioral changes. This finding could be explained by the probability of roflumilast to cause some psychiatric symptoms such as anxiety, depression, and insomnia which was proved to be two to three times as common in the roflumilast groups as in the placebo groups.

***Reference:***

Gupta, S. (2012). Side-effects of roflumilast. *The Lancet*, *379*(9817), 710-711.

**7- Total number of crossed square not found in figure.**

**Response**:

A Figure of total number of crossed square has been added to the figures file

**8- Page 18: Any explanation about this effect.**

**Response**:

Although roflumilast caused non-significant change of biochemical markers in positive control rats, it may cause some behavioral changes. This finding could be explained by the probability of roflumilast to cause some psychiatric symptoms such as anxiety, depression, and insomnia which was proved to be two to three times as common in the roflumilast groups as in the placebo groups.

***Reference:***

Gupta, S. (2012). Side-effects of roflumilast. *The Lancet*, *379*(9817), 710-711.

**9-Results about PDE level not explained in this result section.**

**Response**:

Results of hippocampal PDE-4 gene expression is explained in the revised manuscript

**10- Page 26: neurotrophic**

**Response**

Corrected in the revised manuscript

**11- Page 27: BDNF**

**Response**

Corrected in the revised manuscript

**12- Page 44: Please changes the group legend, between positive control and depressed groups.**

**Response**

Corrected in the figures file and in the revised manuscript and a figure of total number of crossed squares was added

**13- Page 45: please arrange back the figure for easier to capture the results.**

**Response**

Figures are rearranged for better interpretation in the figures file
